# MCL1 promotes porcine epidemic diarrhea virus replication by modulating arachidonic acid metabolic pathway

**DOI:** 10.1371/journal.ppat.1014170

**Published:** 2026-04-24

**Authors:** Hongqi Shang, Shanshan Yang, Min Sun, Yongxiang Zhao, Rongli Guo, Wei Wang, Bingxu Qian, Yunchuan Li, Mi Hu, Xianyu Bian, Qiuxia Cao, Chengcheng Li, Baochao Fan, Bin Li

**Affiliations:** 1 Institute of Veterinary Medicine, Jiangsu Academy of Agricultural Sciences, Nanjing, China; 2 Key Laboratory of Veterinary Biological Engineering and Technology, Ministry of Agriculture, Nanjing, China; 3 Jiangsu Key Laboratory for Food Quality and Safety-State Key Laboratory Cultivation Base of Ministry of Science and Technology, Nanjing, China; 4 Jiangsu Co-innovation Center for Prevention and Control of Important Animal Infectious Diseases and Zoonoses, Yangzhou, China; 5 GuoTai (Taizhou) Center of Technology Innovation for Veterinary Biologicals, Taizhou, China; 6 School of Life Sciences, Jiangsu University, Zhenjiang, China; 7 School of Food and Biological Engineering, Jiangsu University, Zhenjiang, China; Spanish National Center for Biotechnology (CNB-CSIC), SPAIN

## Abstract

Porcine epidemic diarrhea virus (PEDV) poses a significant threat to the global swine industry; however, the host factors that support its replication remain poorly understood. Our previous study showed that myeloid cell leukemia 1 (MCL1) is a pro-PEDV replication cellular factor through genome-scale CRISPR-Cas9-knockout (KO) screening. Nevertheless, the molecular mechanism whereby MCL1 promotes PEDV replication is unclear. In this study, we first demonstrated that MCL1 promotes PEDV replication through its BCL-2 homology (BH) domain. Deletion of MCL1 prevented arachidonic acid (AA) from undergoing β-oxidation which led to the increase of free AA and activation of its secondary metabolic pathways resulting in significant inhibition of PEDV replication. Complementation of MCL1-KO cells with a BH domain fragment of MCL1 restored β-oxidation capacity and rescued PEDV replication. In addition, we identified acyl-CoA synthetase bubblegum family member 1 (ACSBG1) as a novel metabolic regulator that binds to the N-terminus of MCL1, rather than its BH domain, and cooperates with MCL1 to facilitate AA β-oxidation. We further demonstrated that ACSBG1 and MCL1 act together as proviral factors specifically during the replication stage of PEDV infection. In summary, this work reveals a unique and concerted interaction between MCL1 and ACSBG1 that function together to promote PEDV replication by regulating the AA metabolic pathway.

## Author summary

PEDV is a highly infectious enteropathogenic coronavirus that imposes an enormous economic burden on the global swine industry. Despite the ongoing identification of host factors involved in the viral life cycle, the mechanisms how these host factors orchestrate PEDV replication have not been fully understood. In this study, we identified the host protein MCL1 as a pivotal proviral factor for PEDV replication and elucidated its underlying molecular mechanism. Briefly, Our results unveil a dynamic intracellular mechanism whereby ACSBG1 interacts with the N-terminus of MCL1 to cooperatively activate the AA metabolic pathway. Specifically, the BH3 domain of MCL1 facilitates mitochondrial β-oxidation of AA, fueling the energetic demands of PEDV replication. Our findings delineate a novel role for MCL1 in the AA metabolic pathway and provide critical insights into the crosstalk between long-chain unsaturated fatty acid metabolism and PEDV infection.

## 1. Introduction

Porcine epidemic diarrhea (PED) is a highly prevalent and virulant porcine intestinal disease caused by porcine epidemic diarrhea virus (PEDV). The mortality rate of PED can reach up to 100% in suckling piglets, therefore, poses substantial economic damage on the global swine industry [1,2]. As a member of the Alphacoronavirus genus, PEDV was initially reported in the UK in the 1970s and was prevalent throughout Europe, Asia, and the Americas [3–5]. In 2010, a highly pathogenic PEDV variant appeared in China and spread quickly worldwide [5–7]. Conventional PED vaccines failed to provide sufficient protection for piglets against the PEDV variant [8,9]. Despite advancements in vaccine technology, the variant strains continue to emerge with accelerated rate and are more virulent, pathogenic, and contagious. Because of vaccine immunologic failure, increased mortality and secondary infections, PEDV prevention and control become extremely challenging [10–12]. Therefore, identifying host factors essential for PEDV replication is critical not only for elucidating viral pathogenesis but also for discovering novel drug targets for anti-PEDV therapeutics.

Clustered regularly interspaced short palindromic repeats (CRISPR)-CRISPR associated protein 9 (Cas9) system has been explored for pooled genome-scale functional screening. Recently, genome-wide CRISPR knockout (GeCKO) screening has been used to investigate the host factors required for PEDV infection and host factors such as tripartite motif 2 (TRIM2), solute carrier family 35 member A1 (SLC35A1), Protein kinase C θ (PKCθ), interferon-inducible transmembrane protein 3 (IFITM3), Yip family 5 (YIPF5), ST3 beta-galactoside alpha-2,3-sialyltransferase 4 (ST3GAL4), and Ribosomal protein SA (RPSA), were identified to play important roles in the PEDV replication cycle [13–18]. In our prior work, we performed a GeCKO screening using human liver cancer cells (Huh7 cells) with PEDV variant AH2012/12. Deep sequencing analysis of the enriched single guide RNA (sgRNA) population identified several host factors critical for PEDV replication. Further validation studies revealed that loss of myeloid cell leukemia 1 (MCL1) rendered cells resistant to PEDV infection. The purpose of the study was to understand the mechanism of MCL1 in PEDV replication.

As an anti-apoptotic member of the B-cell lymphoma 2 (BCL-2) family, MCL1 is characterized by four BCL-2 homology (BH) domains. The BH3-motif is central to the BH domain’s function, mediating interactions with other BCL-2 family representatives to regulate intrinsic apoptosis [19]. MCL1 is distinguished from its homologs by a unique N-terminal region that confers dynamic regulation. This region harbors a mitochondrial targeting signal (MTS), proline-glutamic acid-serine-threonine (PEST)-rich sequences, and multiple sites for post-translational modifications (PTMs) such as ubiquitination and phosphorylation [20]. Furthermore, the C-terminal transmembrane (TM) domain anchors MCL1 to lipid membranes, predominantly the outer mitochondrial membrane (OMM) [21]. Collectively, MCL1 is involved in a wide array of cellular functions including but are not limited to cellular differentiation, cell cycle progression, DNA damage response (DDR), autophagy, mitochondrial dynamics, calcium handling, and metabolism [22]. Indeed, numerous reports have demonstrated that MCL1 plays important roles in viral infections. Currently, MCL1 modulation of viral replication through apoptosis is most extensively studied [23]. However, the role of MCL1 in viral infection through the non-apoptotic signaling pathways begin to be appreciated. Thus, it is necessary to explore the different regulatory roles of MCL1 to identify which specific role contributes to its promotion of PEDV replication.

In this study, we identified the MCL1 BH domain that modulated PEDV replication. Transcriptome sequencing results showed that deletion of MCL1 prevented AA from undergoing β-oxidation and shunted AA to secondary metabolic pathways which inhibited PEDV replication. During the process, we identified a novel metabolic regulator that was closely associated with PEDV infection, acyl-CoA synthetase bubblegum family member 1 (ACSBG1). ACSBG1 interacted with N-terminus of MCL1 to synergistically facilitate mitochondrial β-oxidation of AA. Altogether, these findings revealed that the AA metabolic pathway co-regulated by MCL1 and ACSBG1 played important role in PEDV infection. These findings may provide new anti-PEDV targets for therapeutic drug development.

## 2. Results

### 2.1 MCL1 is an essential host factor for PEDV replication

Building on our previous genome-scale CRISPR-Cas9 knockout screening that identified MCL1 as a pro-viral host factor for PEDV infection in Huh7 cells [24], we first confirmed this finding via targeted gene knockout. Indeed, the PEDV replication was significantly inhibited in the stable MCL1-KO Huh7 cells (S1A–S1D Fig).

**Fig 1:**
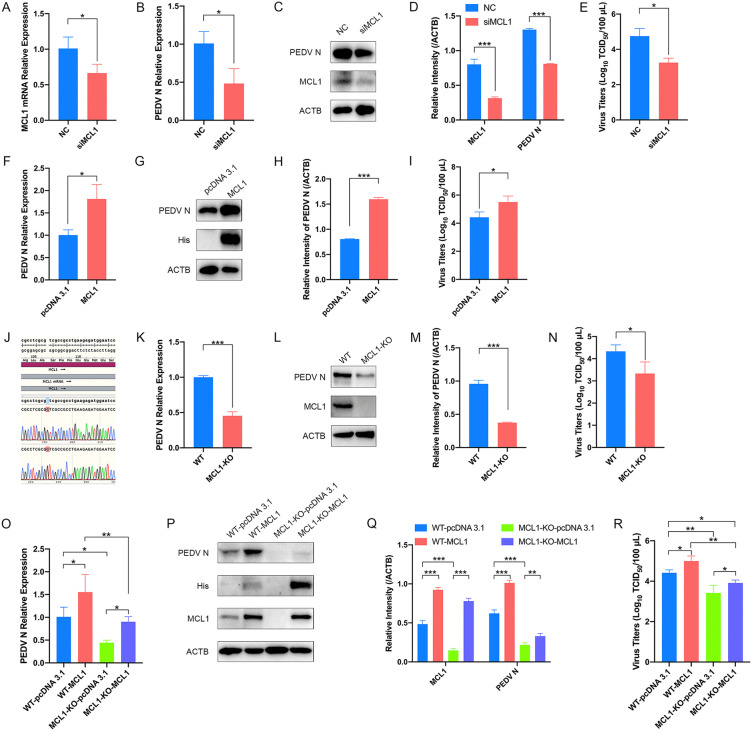
MCL1 acts as a key host factor facilitating PEDV infection. **(A to E)** The effect of MCL1 knockdown on PEDV infection. LLC-PK1 cells were transfected with siRNA against MCL1 or with control siRNA (NC) and were then infected with PEDV (AH2012/12, multiplicity of infection (MOI) = 0.1) for 24 h. Cell samples were harvested and subjected to quantitative real-time PCR (qRT-PCR) to evaluate relative MCL1 **(A)** and PEDV N **(B)** mRNA expression levels. Western blot was applied to detect the expression levels of MCL1 and PEDV N proteins **(C)** with ACTB as a loading control. Quantitative comparisons of MCL1 and PEDV N proteins were analyzed by gray intensity scanning of blots using ImageJ software **(D)**. Cell culture supernatants were also collected, and viral titers **(E)** were determined by limiting serial dilutions. **(F to I)** The effect of MCL1 overexpression on PEDV infection. LLC-PK1 cells were transfected with pcDNA 3.1-MCL1-His plasmid or empty vector for 12 h and then infected with PEDV (AH2012/12, MOI = 0.1). At 24 hpi, the cell lysates were subjected to qRT-PCR **(F)** and western blot **(G)**. The band intensity was quantified using ImageJ software **(H)**. Culture supernatants were collected and the viral titers were detected by TCID_50_ assays **(I)**. **(J)** Validation of the MCL1-KO LLC-PK1 cell line by gDNA sequencing. **(K to N)** The effect of MCL1-KO in LLC-PK1 cells on PEDV infection. The MCL1-KO and WT LLC-PK1 cells were infected with PEDV (AH2012/12, MOI = 0.1) for 24 h. The samples from different groups were harvested and subjected to qRT-PCR **(K)**, western blot **(L and M)**, and TCID_50_ assays **(N)** to determine mRNA relative expression and protein levels of PEDV N, MCL1 protein expression level, and viral titers, respectively. **(O to R)** The effect of MCL1 overexpression in WT and MCL1-KO cells on PEDV infection. The MCL1-KO and WT LLC-PK1 cells were transfected with pcDNA 3.1-MCL1-His plasmid or empty vector for 12 h and then infected with PEDV (AH2012/12, MOI = 0.1). At 24 hpi, the samples were collected and subjected to qRT-PCR **(O)**, western blot **(P and Q)**, and TCID_50_ assays **(R)**. The presented results represent the means and standard deviations of the data from three independent experiments. *, *P* < 0.05; **, *P* < 0.01; ***, *P* < 0.001.

To extend this finding to a more physiologically relevant porcine system, we selected LLC-PK1 cells, which exhibited the highest endogenous MCL1 expression among several susceptible cell lines (S1E–S1G Fig). Functional analyses in LLC-PK1 cells further demonstrated that MCL1 protein promotes the replication of PEDV. Specifically, transient siRNA-mediated depletion of MCL1 suppressed the transcription and protein levels of PEDV N (Fig 1A–1D), whereas ectopic expression of MCL1 enhanced those levels of PEDV N (Fig 1F–1H). Similarly, the viral titers of PEDV were markedly reduced in MCL1 knockdown cells while those were significantly increased in MCL1 overexpressed cells (Fig 1E and 1I).

To definitively establish this pro-viral role, we generated a stable MCL1-KO LLC-PK1 cell line, which were validated by gDNA sequencing (Fig 1J). Consistent with our transient knockdown results, stable knockout of MCL1 severely impaired PEDV replication (Fig 1K–1N). Crucially, this replication defect was partially restored by the supplementation of MCL1 expression vectors in the KO cells (Fig 1O–1R), unequivocally demonstrating its essential function in promoting viral replication.

### 2.2 MCL1-KO inhibits the early- and late- stage replication of PEDV infection cycle

**Fig 2:**
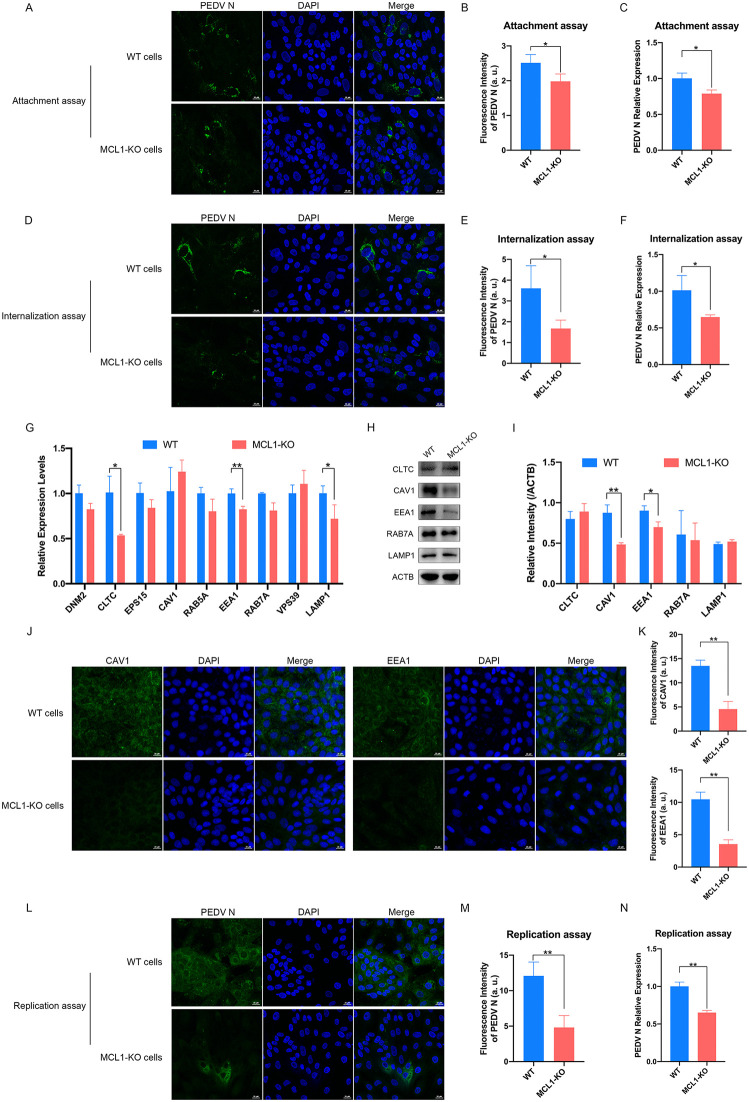
MCL1-KO inhibits the early- and late- stage replication of PEDV infection cycle. For attachment assay, MCL1-KO and WT cells were inoculated with PEDV (AH2012/12, MOI = 5) at 4 °C for 1 h and washed with phosphate buffer saline (PBS) to remove most unattached virions. The cells were fixed and used laser confocal imaging was used to detect PEDV N protein **(A)**. Relative fluorescence intensity was quantified using ImageJ software and is presented in arbitrary units (a. u.) for comparison among different groups **(B)**. Total RNAs were extracted from cells and subjected to qRT-PCR to determine PEDV N mRNA relative expression **(C)**. For internalization assay, MCL1-KO and WT cells were inoculated with PEDV (AH2012/12, MOI = 5) at 4 °C for 1 h, washed with PBS, and subsequently incubated at 37 °C for 1 h. The PEDV N protein levels **(D)** and their fluorescence intensity **(E)** and the PEDV N mRNA relative expression **(F)** were conducted to evaluate the internalization of PEDV. The expression levels of genes related to endocytosis pathways in WT and MCL1-KO cells were detected via qRT-PCR **(G)** and western blot **(H)**. The band intensity of the indicated protein was quantified using ImageJ software **(I)**. The expression levels of CAV1 and EEA1 in WT and MCL1-KO cells were also evaluated by indirect immunofluorescence assays (IFA) **(J)**, and the fluorescence intensity (a. u.) was measured using ImageJ software **(K)**. For replication assay, MCL1-KO and WT cells were transfected with infectious PEDV (AH2012/12)-BAC plasmid. After 6 hours, the washed cells were maintained in fresh serum-free DMEM containing 7.5 μg/mL trypsin for 48 h. The PEDV N protein signals **(L)** and their fluorescence intensity **(M)** and PEDV N mRNA relative expression **(N)** in MCL1-KO and WT cells were measured. Scale bars = 20 μM. The presented results represent the means and standard deviations of the data from three independent experiments. *, *P* < 0.05; **, *P* < 0.01.

To explore the role of MCL1 in different stages of the PEDV infection cycle, we performed attachment and internalization assays following previously described protocols [25,26]. We found that the deletion of MCL1 could significantly reduce PEDV attachment and entry detected by PEDV N mRNA and N protein signals, although these reductions were relatively small (Fig 2A–2F). Obatoclax, a BH3 domain mimetic antagonist of pan-BCL-2 family of proteins, could decreased the levels of furin expression and thereby inhibited SARS-CoV-2 entry by targeting MCL1 [27]. In combination with the above observations, we hypothesized that the inhibitory effect of MCL1 deletion on PEDV entry may be due to the reduced expression of cell membrane receptors, weakened virion endocytosis, or altered membrane fluidity. Therefore, we further evaluated the expression of genes involved in the PEDV endocytosis pathway [28]. Our results showed that MCL1-KO slightly but significantly reduced the mRNA levels of CLTC, EEA1, and LAMP1 (Fig 2G) and, at the protein level, significantly reduced those of CAV1 and EEA1 (Fig 2H–2K), suggesting impaired PEDV endocytosis in MCL1-KO cells.

Moreover, to distinguish this effect from roles in post-entry events, we bypassed the entry step by transfecting cells with a PEDV infectious clone (PEDV-BAC) [29,30]. In this context, MCL1-KO resulted in a much more pronounced decrease in PEDV N transcript and protein levels (Fig 2L–2N), suggesting that MCL1 had crucial function in PEDV replication phase. Collectively, these data suggest that while MCL1 may contribute to the early stages of viral infection, its primary role is to facilitate a post-entry step, likely PEDV genome transcription and replication.

### 2.3 MCL1 promotes PEDV replication through the BH domain

**Fig 3:**
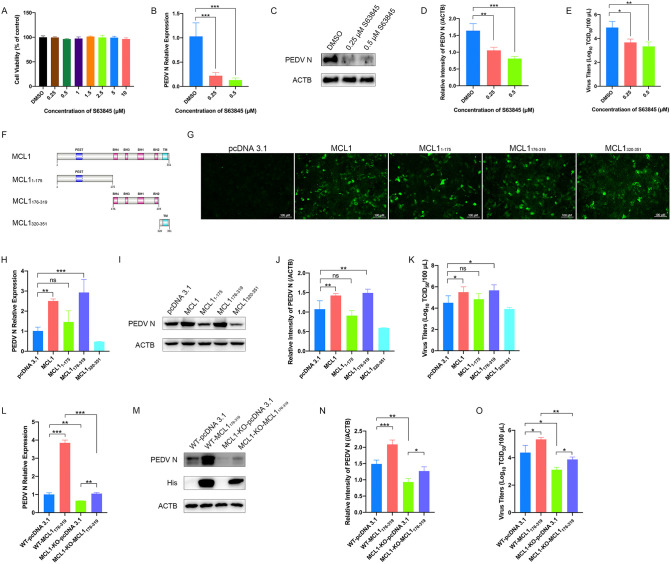
MCL1 promotes PEDV replication through the BH domain. **(A-E)** The effect of S63845 on PEDV infection. LLC-PK1 cells were treated with different concentrations of S63845. After 48 h, cell viability was determined using CCK-8 **(A)**. LLC-PK1 cells were treated with S63845 (0.25 μM and 0.5 μM) and infected with PEDV (AH2012/12, MOI = 0.1) for 24 h, the cells were harvested and subjected to qRT-PCR **(B)** and western blot **(C)** to determine the mRNA relative expression and protein levels of PEDV N. The band intensity of PEDV N was quantified using ImageJ software **(D)**. Cell culture supernatants were collected for TCID_50_ assays **(E)**. The cells treated with DMSO served as the controls. **(F)** Schematic of key truncation constructs of MCL1. **(G)** Verification of overexpression of MCL1 truncations in HEK293T cells. **(H-K)** The effect of MCL1 truncations on PEDV infection. LLC-PK1 cells were transfected with different truncation constructs of MCL1 or empty vector for 12 h and then infected with PEDV (AH2012/12, MOI = 0.1). At 24 hpi, the cell lysates were subjected to qRT-PCR **(H)** and western blot **(I)**. The band intensity of PEDV N was quantified using ImageJ software **(J)**. Culture supernatants were collected for TCID_50_ to detect viral titers **(K)**. **(L-O)** The effect of MCL1_176-319_ overexpression in WT and MCL1-KO cells on PEDV infection. The WT and MCL1-KO LLC-PK1 cells were transfected with pcDNA 3.1-MCL1_176-319_-His plasmid or empty vector for 12 h and then infected with PEDV (AH2012/12, MOI = 0.1). At 24 hpi, the samples were collected and subjected to qRT-PCR **(L)**, western blot **(M and N)**, and TCID_50_ assays **(O)**. The presented results represent the means and standard deviations of the data from three independent experiments. Ns, not significant; *, *P* < 0.05; **, *P* < 0.01; ***, *P* < 0.001.

Next, we investigated the key domain of MCL1 that may involve in PEDV infection. S63845 has been previously demonstrated to be a potent and specific MCL1 inhibitor known to bind its BH3-binding groove with high affinity, thereby disrupting MCL1’s interaction with pro-apoptotic proteins such as BAK and BAX [31]. We initially confirmed that S63845 treatment exhibited minimal cytotoxicity in LLC-PK1 cells at concentrations up to 10 μM (Fig 3A). Treatment with 0.25 μM and 0.5 μM S63845 resulted in a significant reduction in both PEDV N expression levels and viral titers in LLC-PK1 cells (Fig 3B–3E).

To further validate the functional importance of the BH domain, we engineered a series of plasmids expressing truncated forms of MCL1 (Fig 3F), which comprised the N-terminal domain (MCL1_1–175_), the BH domain (MCL1_176–319_), and the transmembrane domain (MCL1_320–351_). Following validation of their expression in HEK293T cells (Fig 3G), these constructs were transfected into LLC-PK1 cells prior to PEDV infection. Notably, MCL1_176–319_ was able to promote PEDV replication at the same level as the full-length MCL1 while other truncated versions of MCL1 had no significant effects on viral replication (Fig 3H–3K). Moreover, ectopic expression of the BH domain (MCL1_176–319_) in MCL1-KO cells successfully restored PEDV replication, highlighting the crucial role of the BH domain in MCL1-mediated effects on PEDV infection (Fig 3L–3O).

### 2.4 MCL1-KO contributes to the abnormal AA metabolism within cells

Given that BCL-2 family proteins are established regulators of programmed cell death (PCD) via interactions mediated by their BH domain [32], we initially postulated that MCL1 promoted PEDV replication through the BH domain-dependent regulation of PCD. We used a series of commercial inhibitors targeting apoptosis (Z-VAD-FMK), ferroptosis (Ferrostatin-1, Fer-1), and autophagy (3-MA) to assess their impact on PEDV infection in MCL1-KO cells. However, inhibition of these pathways failed to rescue the impaired viral replication observed in MCL1-KO cells, collectively indicating that PCD is not the primary mechanism involved in MCL1-mediated PEDV infection (S2A and S2B Fig).

**Fig 4:**
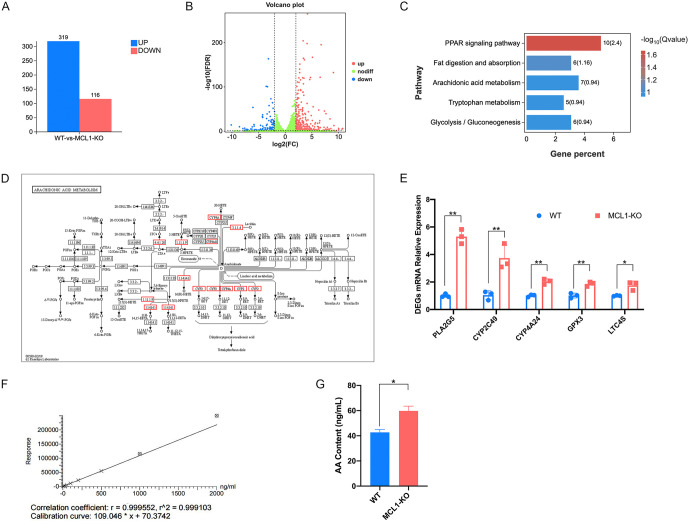
MCL1-KO contributes to the abnormal AA metabolism within cells. **(A-D)** Transcriptomic analysis of DEGs between WT and MCL1-KO LLC-PK1 cells. Compared to WT cells, the total numbers **(A)** and fold changes **(B)** of upregulated and downregulated DEGs were selected with FDR < 0.05 and fold change ≥ 4 or ≤ -4. DEGs were subjected to KEGG enrichment analysis and the top five signaling pathways are presented **(C)**. The AA metabolism pathway derived from the KEGG database was shown **(D)**, and the upregulated DEGs enriched in the AA metabolism pathway were enclosed in red boxes. The DEGs enriched in AA metabolism pathway were validated by qRT-PCR **(E)**. **(F and G)** The AA contents in MCL1-KO and WT LLC-PK1 cells were measured by LC-MS analysis. AA (standard) was serially diluted to generate a standard curve **(F)**. MCL1-KO and WT LLC-PK1 cells were harvested respectively and the AA contents were quantified based on the standard curve **(G)**. The presented results represent the means and standard deviations of the data from three independent experiments. *, *P* < 0.05; **, *P* < 0.01; ***, *P* < 0.001.

Since the regulation of PCD did not account for its pro-viral function, we explored alternative mechanisms, acknowledging that the MCL1 BH domain also modulates other critical cellular processes, including lipid metabolism, Ca^2+^ homeostasis, and redox balance [33]. To further identify the mechanism underlying MCL1-promoted PEDV infection and the key pathway regulated by the BH domain, we performed RNA sequencing (RNA-seq) on total RNA isolated from WT and MCL1-KO LLC-PK1 cells. Compared to the WT cells, MCL1-KO cells showed a total of 435 differentially expressed genes (DEGs), comprising 319 upregulated and 116 downregulated transcripts (false discovery rate (FDR) of < 0.05 and fold change of ≤ -4 or ≥ 4) (Fig 4A and 4B). Kyoto Encyclopedia of Genes and Genomes (KEGG) pathway analysis revealed significant enrichment of the peroxisome proliferator-activated receptor (PPAR) signaling pathway, the fat digestion and absorption pathway, and the AA metabolism pathway following MCL1-KO (Fig 4C). The DEGs within these pathways are predominantly associated with the uptake, transport, activation, and catabolism of unsaturated fatty acids. It is worth noting that the AA metabolism pathway displayed the highest density of DEGs compared with the PPAR signaling and fat digestion and absorption pathway (Figs 4D, S3A and S3B). Previous studies have demonstrated that AA and its secondary metabolites modulated nuclear receptors such as PPARs and fibrotic pathways in kidney diseases [34]. Combining with our findings, we hypothesize that the abnormal AA metabolism caused by MCL1 deficiency triggers the activation of the PPAR pathway and changes in the fatty acid digestion and absorption pathway.

To validate this hypothesis, we first detected the expression of key DEGs in the AA metabolism pathway, including phospholipase A2 Group V (PLA2G5), cytochrome P450 2C49 (CYP2C49), cytochrome P450 4A24 (CYP4A24), glutathione peroxidase 3 (GPX3), and leukotriene-C4 synthase (LTC4S), by qRT-PCR, which affirmed the reliability of the transcriptomic results (Fig 4E). The upregulation of these genes strongly implied a potential accumulation of the substrate AA (due to increased PLA2G5 expression) and its downstream metabolites, such as leukotrienes (e.g., LTC4, via LTC4S) and eicosanoids (e.g., EETs and HETEs, via CYP families) [35,36]. Therefore, we proceeded to directly quantify the central metabolite, AA. After establishing a standard curve (Fig 4F), we measured cellular AA content in WT and MCL1-KO cells via liquid chromatography-mass spectrometry (LC-MS). Consistent with our hypothesis, the AA content was significantly elevated in MCL1-KO cells compared to that in WT cells (Fig 4G).

### 2.5 AA and its secondary metabolites can inhibit PEDV replication

**Fig 5:**
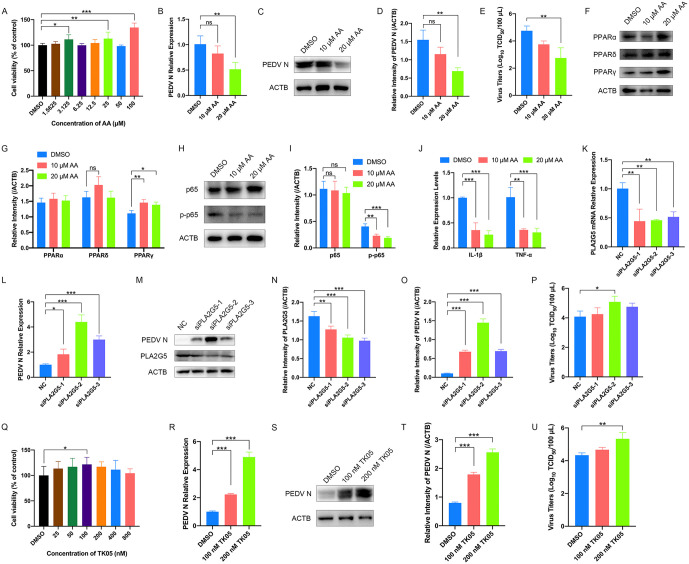
AA and its secondary metabolites can inhibit PEDV replication. **(A-J)** The effect of AA on PEDV infection. LLC-PK1 cells were treated with gradient concentrations of AA for 48 h. Cell viability was determined using CCK-8 **(A)**. LLC-PK1 cells were treated with AA (10 μM and 20 μM) and infected with PEDV (AH2012/12, MOI = 0.1) for 24 h, the cells were harvested and subjected to qRT-PCR **(B)** and western blot **(C)**. The band intensity of PEDV N was quantified using ImageJ software **(D)**. The cell supernatants were collected for TCID_50_ assays **(E)**. Protein levels of PPARα, PPARδ, and PPARγ were analyzed by western blotting **(F)**, with densitometric quantification shown in **(G)**. Total and phosphorylated p65 levels were analyzed by western blotting **(H)**, with ImageJ-based quantification shown in **(I)**. The mRNA levels of IL-1β and TNF-α were measured by qRT-PCR **(J)**. The cells treated with DMSO served as the controls. **(K-P)** The effect of PLA2G5 knockdown on PEDV infection. LLC-PK1 cells were transfected with siRNA targeting PLA2G5 or control siRNA and then infected with PEDV strain AH2012/12 (MOI = 0.1) for 24 h. Cells were harvested and subjected to qRT-PCR **(K and L)** and western blotting **(M)** to assess PLA2G5 and PEDV N expression at the mRNA and protein levels. Band intensities of PLA2G5 **(N)** and PEDV N **(O)** were quantified using ImageJ. Viral titers in cell supernatants were determined by TCID_50_ assays **(P)**. **(Q-U)** The effect of TK05 on PEDV infection. Cell viability of LLC-PK1 cells was measured after treatment with different concentrations of TK05 for 48 hours **(Q)**. LLC-PK1 cells were treated with AA (100 nM and 200 nM) and infected with PEDV (AH2012/12, MOI = 0.1) for 24 h, the cells were harvested and subjected to qRT-PCR **(R)** and western blot **(S)**. The band intensity of PEDV N was quantified using ImageJ software **(T)**. The cell supernatants were collected for TCID_50_ assays **(U)**. The cells treated with DMSO served as the controls. The presented results represent the means and standard deviations of the data from three independent experiments. *, *P* < 0.05; **, *P* < 0.01; ***, *P* < 0.001; ns, not significant.

Based on the preceding findings, we hypothesized that the impaired PEDV replication in MCL1-KO cells was mediated by the sustained accumulation of AA. To test this hypothesis, we first evaluated the effects of exogenous AA on PEDV infection in LLC-PK1 cells. A CCK-8 assay established a non-toxic concentration range, demonstrating that AA concentrations up to 50 μM did not adversely affect cell viability; notably, a concentration of 100 μM even promoted cell proliferation (Fig 5A). Consistent with our hypothesis, treatment with 20 μM AA significantly suppressed both PEDV N expression and viral titers (Fig 5B–5E). As described in Section 2.4, cells with elevated free AA levels may trigger the activation of the PPAR pathway. Thus, we investigated whether AA exerts its anti-PEDV activity through PPAR signaling. Our results showed that AA treatment markedly increased PPARγ expression and reduced phospho-p65 (p-p65) levels, whereas total p65 abundance remained unchanged, indicating suppression of NF-κB activation (Fig 5F–5I). In addition, AA significantly decreased the mRNA levels of IL-1β and TNF-α (Fig 5J). Taken together, these findings suggest that AA inhibit PEDV replication by suppressing the PEDV-induced inflammatory environment, potentially through the PPARγ-NF-κB axis.

To complement our findings and further investigate the role of endogenous AA, we targeted PLA2G5, a key member of the sPLA2 family involved in the release of AA from membrane phospholipids [37]. We employed siRNA to silence PLA2G5 expression, thereby reducing endogenous AA release. The knockdown efficiency was confirmed by a significant reduction in PLA2G5 mRNA levels in the siPLA2G5 group as compared to the non-targeting control (siNC) group (Fig 5K). Depletion of endogenous AA via PLA2G5 silencing significantly increased PEDV N expression and viral titers compared with those in the siNC-treated group (Fig 5L–5P). These findings more closely reflect the physiological changes occurring in cells, where MCL1-KO markedly increases PLA2G5 expression, resulting in sustained release of AA and thereby mediating antiviral activity.

Given our previous analysis suggesting the potential elevation of secondary metabolites such as LTC4 in MCL1-KO cells, we sought to determine whether LTC4 exerts anti-PEDV effects. Given the lack of a standard for LTC4, we inhibited LTC4 synthesis using TK05, a selective inhibitor of LTC4S, the enzyme responsible for LTC4 production. After confirming that TK05 was non-cytotoxic below 800 nM (Fig 5Q), we treated cells with 100 nM and 200 nM TK05 in the subsequent experiments, concentrations informed by prior studies [38]. We found that both TK05 concentrations significantly increased PEDV N expression levels (Fig 5R–5T) and led to a corresponding significant rise in viral titers in the supernatant compared to DMSO-treated controls (Fig 5U). Collectively, our results demonstrate that the antiviral activity of AA and its downstream metabolite LTC4 likely represents a major mechanism by which MCL1-KO suppresses PEDV replication.

### 2.6 MCL1 interacts with ACSBG1 to promote AA metabolism

**Fig 6:**
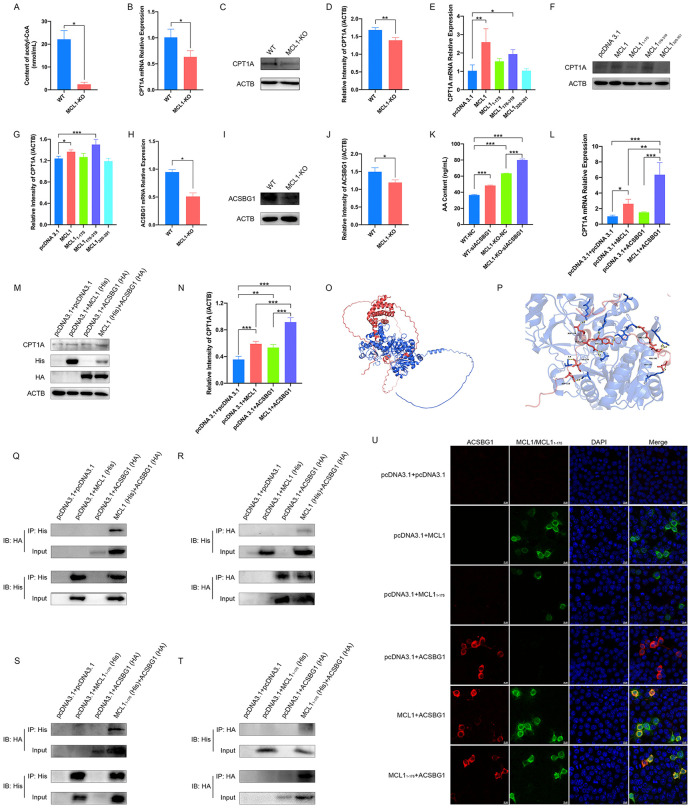
MCL1 interacts with ACSBG1 to promote AA β-oxidation. **(A-D)** The effect of MCL1-KO on energy metabolism. The levels of acetyl-CoA in MCL1-KO and WT LLC-PK1 cells were measured using a colorimetric assay **(A)**. qRT-PCR **(B)** and western blot **(C)** were performed to evaluate the mRNA and protein levels of CPT1A in MCL1-KO and WT LLC-PK1 cells. Quantitative comparisons of CPT1A were analyzed by gray intensity scanning of blots **(D)**. MCL1-KO cells were transfected with different truncation constructs of MCL1 or empty vector for 24 h. The cell lysates were collected and subjected to qRT-PCR **(E)** and western blot **(F)**. The band intensity of CPT1A was quantified using ImageJ software **(G)**. ACSBG1 mRNA relative expression and protein levels in MCL1-KO and WT LLC-PK1 cells were measured by qRT-PCR **(H)** and western blot **(I)**. The band intensity of ACSBG1 was quantified using ImageJ software **(J)**. WT and MCL1-KO LLC-PK1 cells were transfected with siRNA against ACSBG1 or with NC, and harvested after 24 h to measure AA content **(K)**. MCL1-KO cells were transfected with MCL1 and ACSBG1, MCL1 alone, ACSBG1 alone, or empty vector, ensuring an equal total amount of plasmids in each group. After 24 hours, the cell lysates were collected and subjected to qRT-PCR **(L)** and western blot **(M)**. The band intensity of CPT1A was quantified using ImageJ software **(N)**. Ribbon diagram represented the structure of porcine-derived ACSBG1-MCL1 complex with ACSBG1 colored in blue and MCL1 in red **(O)**. Close-up view of the interface highlighted specific residues from ACSBG1 (blue) and MCL1 (red), and the predicted interaction sites were indicated by yellow bonds **(P)**. **(Q-T)** The interaction between ACSBG1 and MCL1. HEK293T cells were cotransfected with pcDNA3.1-MCL1-His and pcDNA3.1-ACSBG1-HA plasmids. After 36 h, the cells were lysed and subjected to co-IP using anti-His binding beads. Precipitated proteins were analyzed by western blot with an anti-HA antibody **(Q)**. Cell lysates immunoprecipitated with anti-HA binding beads were analyzed by western blot with an anti-His antibody **(R)**. HEK293T cells transfected with corresponding empty vectors served as the controls. ACSBG1-HA were immunoprecipitated with anti-His binding beads **(S)** and MCL1_1-175_-His were immunoprecipitated with anti-HA binding beads **(T)**. HEK293T cells transfected with corresponding empty vectors served as the controls. HEK293T cells were cotransfected with the pcDNA 3.1-MCL1/MCL1_1-175_-His and pcDNA 3.1-ACSBG1-HA plasmids. After 36 h, the cells were fixed to detect the localization of ACSBG1-HA and MCL1/MCL1_1-175_-His using a laser confocal microscope using anti-His and -HA antibodies **(U)**. HEK293T cells transfected with corresponding empty vectors served as the controls. The presented results represent the means and standard deviations of the data from three independent experiments. *, *P* < 0.05; **, *P* < 0.01; ***, *P* < 0.001.

Under physiological conditions, mitochondrial β-oxidation is the primary pathway for fatty acid catabolism and energy production. However, despite no observed upregulation of β-oxidation pathways in our previous transcriptomic data, we noted a significant enrichment of monooxygenase activity in the Gene Ontology (GO) analysis (S3C Fig). This indicates an activation of the CYP450 monooxygenase pathway for AA metabolism, suggesting a shift towards ω-oxidation – a compensatory metabolic pathway often engaged during fatty acid overload or compromised mitochondrial β-oxidation. Given that mitochondrial matrix-localized MCL1 has been shown to modulate long-chain fatty acid oxidation (FAO) through direct interaction with very long chain acyl-CoA dehydrogenase (VLCAD) via its BH3 domain [39], we postulated that MCL1-KO led to impaired mitochondrial β-oxidation, resulting in AA accumulation and the activation of compensatory metabolic pathways. Hence, we first measured intracellular acetyl-CoA levels, observing a significant decrease in MCL1-KO cells compared to WT (Fig 6A). Furthermore, the expression of carnitine palmitoyltransferase 1a (CPT1A), a rate-limiting enzyme in mitochondrial β-oxidation [40], was significantly downregulated in MCL1-KO cells (Fig 6B–6D). These findings collectively suggested that MCL1 deletion impairs AA β-oxidation, thereby reduces intracellular ATP supply. Subsequently, we explored the key domains of MCL1 that regulate β-oxidation. As shown in Fig 6E–6G, the expression levels of CPT1A were significantly increased after transfecting with MCL1 or MCL1_176–319_ in MCL1-KO cells, indicating the promoting effect of the MCL1 BH3 domain on β-oxidation.

Prior to mitochondrial β-oxidation, fatty acids must be activated into fatty acyl-CoAs by long-chain acyl-CoA synthetases (ACSLs), a prerequisite for their transport across the outer mitochondrial membrane. While ACSL4 is known to preferentially metabolize polyunsaturated fatty acids (PUFAs) like AA and docosahexaenoic acid [41,42], its expression remained unchanged at both mRNA and protein levels following MCL1-KO (S4A–S4C Fig). To identify alternative ACS family members involved, we screened for DEGs based on an FDR < 0.05 and an absolute log2FC ≥ 2 (S4D Fig). Instead of ACSL4, we identified ACSBG1 as a novel metabolic regulator whose expression was significantly downregulated after MCL1-KO (Fig 6H–6J). To determine whether ACSBG1 is involved in the AA metabolism regulated by MCL1, ACSBG1 was further knocked down in MCL1-KO cells using specific siRNA (siACSBG1), which led to a significant elevation in intracellular AA content compared to that in MCL1-KO cells transfected with NC or in WT cells transfected with siACSBG1 alone (Fig 6K). Based on this, we evaluated the effect of ACSBG1 on MCL1-mediated β-oxidation. Our results showed that cotransfection of MCL1 and ACSBG1 markedly increased the expression of CPT1A at both the mRNA and protein levels in MCL1-KO cells (Fig 6L–6N). These results suggested a synergistic role for ACSBG1 and MCL1 in the regulation of AA β-oxidation.

Given the observed association between MCL1 and ACSBG1, computational modeling was employed to predict the interaction and their binding sites between porcine ACSBG1 and porcine MCL1 (Fig 6O and 6P). Co-immunoprecipitation (co-IP) assays were further performed to investigate their direct physical interaction. Our results demonstrated that HA-ACSBG1 was robustly co-immunoprecipitated with His-MCL1; conversely, His-MCL1 was reciprocally co-immunoprecipitated with HA-ACSBG1, with no signal detected in respective control immunoprecipitations (Fig 6Q and 6R). Further supporting a physical interaction, laser confocal microscopy (LCM) revealed strong cytoplasmic co-localization between HA-ACSBG1 and His-MCL1 (Fig 6U). To delineate the specific MCL1 domains mediating this interaction, we assessed the interaction between ACSBG1 and MCL1 truncations. Referring to Fig 6P, all predicted potential ACSBG1 binding sites on MCL1 were exclusively localized within MCL1_1–175_, with no significant binding sites identified in MCL1_176–319_. Consistent with the results generated from computational modelings, co-IP assays showed a robust interaction between HA-ACSBG1 and the N-terminal fragment, His-MCL1_1–175_ (Fig 6S and 6T). Colocalization analysis by LCM further confirmed cytoplasmic co-localization of HA-ACSBG1 with His-MCL1_1–175_ (Fig 6U).

However, there have been reports that ACSL1 interacts with the BH3-binding groove of MCL1 [43], we also examined whether ACSBG1 interacts with the BH domain of MCL1 (MCL1_176–319_). However, no interaction was observed between HA-ACSBG1 and His-MCL1_176–319_ in either direction (S4E and S4F Fig). To assess evolutionary conservation, we examined the interaction and binding domains between human ACSBG1 and human MCL1. Computational predictions showed that human ACSBG1 also interacted with human MCL1 (S4G Fig), but the potential binding sites of human ACSBG1 were identified on PEST-like domain, BH1–3 domain, and transmembrane domain of human MCL1. Notably, the PEST-like domain of human MCL1 exhibited a greater number of potential binding sites and stronger binding affinity for human ACSBG1 compared to the other two domains (S4H–S4J Fig).

Collectively, these comprehensive experimental and computational analyses strongly indicate that ACSBG1 physically interacts with the N-terminus of MCL1, and this interaction likely underlies their cooperative role in regulating AA metabolism.

### 2.7 MCL1 cooperates with ACSBG1 to promote PEDV replication

A previous study demonstrated that PEDV infection induces fatty acid β-oxidation, a process essential for viral replication [44]. Consequently, we proposed that MCL1 exerts its pro-viral effects by promoting intracellular AA β-oxidation to meet the energy demands of viral replication. Given the synergistic role of ACSBG1 and MCL1 in AA metabolism, we investigated whether ACSBG1 plays a comparable role in PEDV infection.

**Fig 7:**
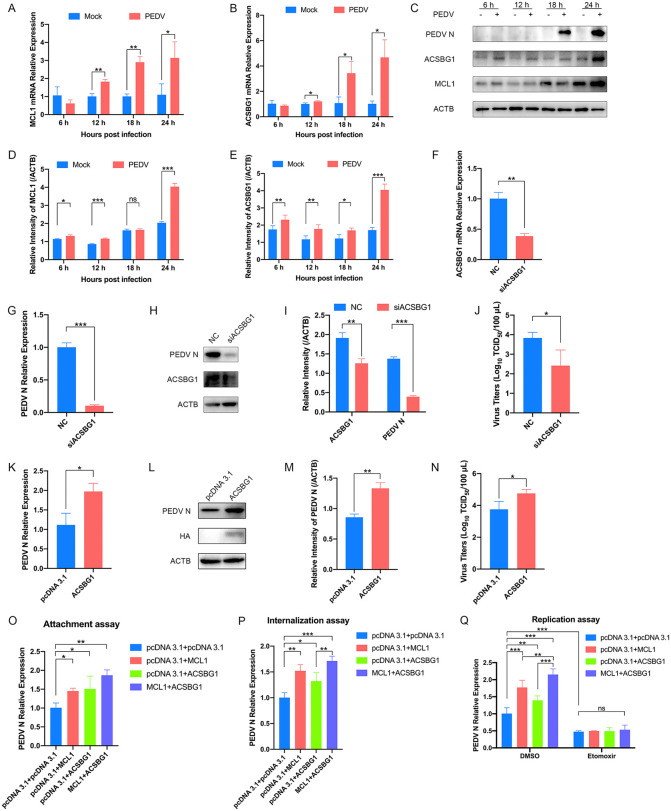
MCL1 cooperates with ACSBG1 to promote PEDV replication. **(A-E)** The dynamic changes of MCL1 and ACSBG1 expression during PEDV infection. LLC-PK1 cells were infected with PEDV (AH2012/12, MOI = 0.1). At 6, 12, 18, and 24 hpi, the cell lysates were collected to detect the mRNA relative expression of MCL1 **(A)** and ACSBG1 **(B)** by qRT-PCR. Western blot was performed to detect the protein expression levels of PEDV N, ACSBG1, MCL1, and ACTB **(C)**. Quantitative comparisons of MCL1 **(D)** and ACSBG1 **(E)** were analyzed by gray intensity scanning of blots. **(F-J)** The effect of ACSBG1 knockdown on PEDV infection. LLC-PK1 cells transfected with siACSBG1 or siNC were infected with PEDV (AH2012/12, MOI = 0.1) for 24 h. Cells were harvested and subjected to qRT-PCR **(F and G)** and western blot **(H and I)** to determine mRNA relative expression and protein levels of ACSBG1 and PEDV N. Cell culture supernatants were also collected for TCID_50_ assays **(J)**. **(K-N)** The effect of ACSBG1 overexpression on PEDV infection. LLC-PK1 cells were transfected with pcDNA 3.1-ACSBG1-HA plasmid or empty vector for 12 h and then infected with PEDV (AH2012/12, MOI = 0.1). At 24 hpi, the cell lysates were subjected to qRT-PCR **(K)** and western blot **(L)**. The band intensity of PEDV N was quantified using ImageJ software **(M)**. Cell supernatants were collected to determine viral titers by TCID_50_ assays **(N)**. **(O-Q)** The effect of ACSBG1 on the PEDV life cycle. LLC-PK1 cells were transfected with plasmids expressing MCL1 and ACSBG1, MCL1 alone, ACSBG1 alone, or the empty vector. Empty vector was added as needed to ensure equal amounts of total plasmid DNA across groups. For attachment assay, LLC-PK1 cells were inoculated with PEDV (AH2012/12, MOI = 5) at 4 °C for 1 h and washed with PBS to remove unattached virions. Total RNA was extracted, and relative PEDV N RNA levels were determined by qRT-PCR **(O)**. For internalization assay, the LLC-PK1 cells were inoculated with PEDV (AH2012/12, MOI = 5) at 4 °C for 1 h, washed with PBS, and subsequently incubated at 37 °C for 1 h. Relative PEDV N RNA levels were measured by qRT-PCR to assess viral internalization **(P)**. For replication assay, LLC-PK1 cells were were pretreated with 200 μm Etomoxir or DMSO and transfected with infectious PEDV (AH2012/12)-BAC plasmid. After 6 hours, the washed cells were maintained in fresh serum-free DMEM containing 7.5 μg/mL trypsin for 48 h. Relative PEDV N RNA levels were then quantified by qRT-PCR **(Q)**. DMSO-treated cells served as vehicle controls. *, *P* < 0.05; **, *P* < 0.01; ***, *P* < 0.001; ns, not significant.

First, we examined the expression kinetics of ACSBG1. Mirroring the pattern observed for MCL1, ACSBG1 mRNA levels exhibited a time-dependent increase, and its protein level was significantly upregulated at late stages of PEDV infection (Fig 7A–7E). Based on these, the functions of ACSBG1 on PEDV infection was further confirmed using loss- and gain-of-function approaches. Knockdown of ACSBG1 significantly suppressed PEDV infection, as evidenced by reduced PEDV N at mRNA and protein levels, and viral titers compared to the siNC group (Fig 7F–7J). Conversely, ACSBG1 overexpression markedly increased PEDV N mRNA expression (Fig 7K), N protein levels (Fig 7L and 7M), and viral titers (Fig 7N). Finally, we examined the contribution of ACSBG1 to the PEDV life cycle. Given the unique role of MCL1 in PEDV infection and its close association with ACSBG1, we transfected LLC-PK1 cells with plasmids expressing ACSBG1, MCL1, or both, while cells transfected with the empty vector served as controls. Consistent with our previous findings, MCL1 overexpression significantly enhanced PEDV attachment and internalization (Fig 7O and 7P). Notably, co-overexpression of ACSBG1 and MCL1 markedly increased PEDV replication compared with overexpression of either ACSBG1 or MCL1 alone, suggesting that ACSBG1 and MCL1 cooperatively promote the replication stage of the PEDV life cycle (Fig 7Q). To further determine whether this proviral effect depends on the regulation of AA β-oxidation, we pretreated LLC-PK1 cells with Etomoxir, a well-established inhibitor of fatty acid β-oxidation [45]. Our results showed that Etomoxir treatment abrogated the proviral effects of MCL1 overexpression, ACSBG1 overexpression, and their co-expression during the PEDV replication stage (Fig 7Q). Overall, these data indicate that MCL1 cooperates with ACSBG1 to promote PEDV replication and that this effect is abolished when cellular β-oxidation is disrupted.

## 3. Discussion

PEDV causes a highly infectious porcine acute intestinal disease, especially with the emerging of the highly lethal variants, bringing enormous burden to the global swine industry. Currently, the veterinary industry lacks effective therapeutic drugs and vaccines for PED, posing significant challenges to its control. Although host factors that contribute to the viral replication are gradually being identified, the mechanism how the host factors regulate the replication process of PEDV has not been fully elucidated. Our previous study identified MCL1 as a key host factor for PEDV replication through GeCKO screening. In this study, we demonstrated in LLC-PK1 cells that MCL1 promoted PEDV replication via its BH domain. Mechanistically, MCL1 deficiency led to aberrant intracellular AA accumulation and activation of downstream secondary metabolic pathways, which in turn suppressed viral propagation. In addition, we characterized ACSBG1 as a novel metabolic regulator that plays an important role in PEDV replication by interacting with the N terminus of MCL1 (residues 1–175), rather than its BH domain, and cooperating with MCL1 to enhance arachidonic acid metabolism, thereby fueling PEDV replication. Taken together, this work revealed a distinct molecular mechanism in which the interaction between ACSBG1 and MCL1 promoted PEDV replication by regulating the AA metabolic pathway. Our findings provided new insights into the crosstalk between long-chain unsaturated fatty acid metabolism and PEDV infection.

We have shown that MCL1 functions as a proviral host factor that acts predominantly at the stage of PEDV replication. Thus, our subsequent experiments focused on elucidating the molecular mechanisms by which MCL1 regulates PEDV replication. Interestingly, we also observed that MCL1-KO inhibited PEDV entry during the attachment and internalization phases. Further analysis of genes involved in endocytic pathways revealed that MCL1-KO markedly reduced the protein levels of CAV1 and EEA1. To our knowledge, a direct link between MCL1 and endocytic pathways has not been reported. Preedakorn et al. showed that CAV1 interacted with MCL1 and prevented it degradation via the ubiquitin-proteasome pathway [46]. Taken together with our findings, we speculate that this may represent a novel mechanism by which MCL1-KO or inhibition of MCL1 restricts infection by viruses that depend on caveolae-mediated endocytosis. Although the mechanism by which MCL1 deficiency impairs PEDV entry was not elucidated specifically in this study, this remains an intriguing topic that warrants detailed investigation in future work.

Multiple studies have reported that MCL1 plays an important role in the replication of various viruses. It has been shown that SARS-CoV-2 N protein specifically interacted with MCL1 PEST motif and recruited a deubiquitinating enzyme USP15 to remove the K63-linked ubiquitination of MCL1, which stabilized this protein, thereby inhibiting apoptosis and promoting the pathogen’s propagation [47]. In our study, ectopic expression of N-terminus of MCL1 (MCL1_1–175_) resulted in a modest but statistically non-significant increase in PEDV N mRNA levels and viral titers. MCL1_1–175_ contains both an MTS and a PEST sequence. The MTS directs MCL1 import into mitochondria, whereas the PEST domain is subject to post-translational modifications that regulate MCL1 turnover through the ubiquitin-proteasome system [48]. Since many viruses could promote their replication by altering the ubiquitination of host proteins [49,50], we speculated that PEDV infection may stabilize MCL1, potentially through viral or virus-induced deubiquitinating activity, thereby prolonging host cell survival and offering a more favorable environment for viral replication. However, this possibility remains to be directly verified. Our results suggest that the BH domain is the key domain underlying the proviral activity of MCL1 during PEDV infection, compared with that of its N-terminus (MCL1_1–175_). Previous studies have implicated MCL1-mediated PCD-related signaling pathways as important regulators during viral infection [51,52]. Considering that this is a canonical function of the MCL1 BH domain, we initially hypothesized that MCL1 might promote PEDV replication by modulating PCD-related pathways. Unexpectedly, pretreatment of LLC-PK1 cells with inhibitors of different types of PCD failed to rescue the reduction in viral loads caused by MCL1-KO. These results lead us to propose a revised hypothesis: with exogenous stimuli such as viral infection, the activation of alternative signaling cascades or recruiting distinct partners could contribute to the pro-viral activity mediated by the MCL1 BH domain.

Evidence from recent literature substantiates our hypothesis. It has been reported that while BCL-2 family homologs, such as BCL-XL and BCL-2, can rescue preimplantation lethality in *Mcl1* ⁻ / ⁻ embryos, they fail to compensate for the metabolic defects observed in later development [53]. In our study, KEGG pathway analysis of transcriptome also suggested that metabolic related pathways were significantly enriched in MCL1-KO LLC-PK1 cells, especially AA metabolism. The qPCR results further verified a significant increase in the mRNA levels of the enzymes that were responsible for AA release and secondary metabolism, indicating a defect in the oxidation of AA, which requires mitochondrial import. Our LC-MS analysis further confirmed a marked increase in free AA levels within MCL1-KO cells, an observation that aligns with reports of lipid accumulation in *Mcl1* ⁻ / ⁻ fasted mice, characterized by an enrichment of long-chain free fatty acids (FFAs), triacylglycerides (TAGs), and cholesterol esters (CEs) [43].

To further clarify the pro-viral role of MCL1 by regulating the AA metabolism, we examined the effect of exogenous and endogenous AA on PEDV replication. We found that supplementing cells with 20 μM AA suppressed PEDV replication, whereas inhibiting AA release in the cytoplasm via knockdown of PLA2G5 enhanced it [54]. We also explored the potential anti-PEDV mechanism of AA in LLC-PK1 cells. We found that AA treatment robustly induced PPARγ expression and concomitantly attenuated activation of the NF-κB pathway, as evidenced by unchanged total p65 levels but reduced p-p65. This shift toward an anti-inflammatory profile was further supported by the marked decrease in IL-1β and TNF-α mRNA levels. Currently, multiple studies have shown that coronavirus infection strongly activates the NF-κB signaling pathway [55,56], and that inhibition of this pathway can suppress viral replication and alleviate coronavirus-mediated cytokine storms and tissue damage [57,58]. In light of the encouraging therapeutic effects of NF-κB inhibitors against SARS-CoV-2, NF-κB is increasingly recognized as a potential druggable target for combating coronavirus infections [59]. Similar therapeutic effects have also been reported in porcine coronavirus infections [60–62]. Therefore, our findings suggest that AA may inhibit PEDV replication by suppressing the PEDV-induced inflammatory environment through the PPARγ/NF-κB axis.

Furthermore, MCL1-KO shunts AA into secondary metabolic pathways leading to the increased synthesis of cyclooxygenase, lipoxygenase, and ω-hydroxylase products [63]. Our transcriptomic data revealed that DEGs were significantly enriched in both the LOX and CYP450 pathways, indicating the potential elevation of secondary metabolites including LTC4, EETs, and HETEs. Thus, we further explored the effect of these secondary metabolites on PEDV replication. Although the absence of an authentic standard for LTC4, together with the lack of standards and selective inhibitors for EETs and HETEs, precluded direct assessment of these metabolites, selective inhibition of LTC4S with TK05 promoted PEDV replication in LLC-PK1 cells. Our results support the notion that increased LTC4 levels, potentially together with other AA metabolites, contribute to the suppression of PEDV replication in MCL1-KO cells. The antiviral effect of different fatty acids has previously been reported in multiple studies. Among these, polyunsaturated fatty acids, such as docosahexaenoic acid (DHA), eicosapentaenoic acid (EPA), and linoleic acid (LA), have been shown to significantly inhibit PEDV replication [64,65]. Together, these observations and our data suggest that AA and its certain downstream metabolites may represent potential therapeutic candidates against PEDV.

As we mentioned earlier, accumulated long-chain fatty acids (LCFAs) and the emergence of compensatory metabolic pathways are often indicative of defective fatty acid β-oxidation (FAO) [66,67]. Consistent with this paradigm, our results demonstrated that FAO activity was significantly attenuated in MCL1-KO LLC-PK1 cells, as evidenced by decreased acetyl-CoA content and CPT1A expression levels. Previous studies by Wright et al. established that MCL1 orchestrates essential steps of mitochondrial long-chain FAO via its interaction with the long-chain acyl-coenzyme A synthetase 1 (ACSL1) [43]. Therefore, we considered whether fatty acid activation mediated by acyl-CoA synthetases might also be compromised under under the conditions of FAO deficiency. Normally, ACSL4 preferentially converts AA into arachidonoyl-CoA (AA-CoA), whereas the ACSL4-(mitochondrial acyl-CoA thioesterase) ACOT2 axis can regenerate free AA within the mitochondrial matrix [68,69]. However, we found that ACSL4 mRNA and protein levels were not significantly changed in LLC-PK1 cells following MCL1-KO. Previous studies suggested that AA generated in the mitochondrial matrix was thought to be used predominantly for mitochondrial membrane phospholipid remodeling rather than serving as a major substrate for β-oxidation [70,71]. In addition, compensatory AA metabolic pathways were activated in MCL1-KO cells. The biosynthesis of their products, including LTC4, EETs, and HETEs, occurs predominantly in membrane systems associated with the nuclear envelope and endoplasmic reticulum rather than in mitochondria [72–74]. Based on these, we hypothesized that the increased AA observed in MCL1-KO cells is more likely to accumulate in the cytosol than in the mitochondrial matrix. Accordingly, other acyl-CoA synthetases may be responsible for cytosolic AA activation, although their contribution appears to be reduced in MCL1-KO cells. Consequently, we screened the transcriptome data for acyl-CoA synthetases whose expression was significantly reduced in MCL1-KO cells and ultimately identified ACSBG1, a member of the acyl-CoA synthetase bubblegum family [75], as a key candidate. Consistent with our expectations, silencing ACSBG1 in MCL1-KO cells led to a marked accumulation of free AA, further confirming the critical role of ACSBG1 in facilitating the mitochondrial import of AA. This finding is also supported by Ye’s report of significantly higher AA levels in the cerebellum of *Acsbg1* ⁻ / ⁻ mice compared to wild-type mice at birth, providing crucial in vivo evidence despite potential age-related changes in AA levels. Additionally, Ye’s study demonstrated that *Acsbg1* ⁻ / ⁻ mice also exhibited altered levels of other specific fatty acids, suggesting that the regulatory scope of ACSBG1 extends beyond AA alone [76].

Given that both ACSBG1 and MCL1 are involved in the regulation of AA metabolism, we hypothesized a direct interplay between these proteins. As anticipated, Co-IP and IFA assays confirmed a physical interaction between ACSBG1 and MCL1. To map the binding interface, we utilized truncation mutants of MCL1. Strikingly, the N-terminal fragment of MCL1 (residues 1–175) retained binding affinity for ACSBG1, whereas the BH domain-containing fragment (residues 176–319) failed to interact. These data diverge from prior reports regarding ACSL1, which interacts with the BH3-binding groove of MCL1, a domain typically occupied by pro-apoptotic molecules [77]. Interestingly, AlphaFold modeling suggested a species-specific variation: human ACSBG1 was predicted to bind multiple domains of MCL1 (N-terminal, BH, and transmembrane), whereas porcine ACSBG1 interacted only with the N-terminus of porcine MCL1. This unique interaction profile implied distinct molecular mechanisms in the porcine host.

Mirroring the pivotal role of MCL1 in PEDV infection, we observed that ACSBG1 expression is significantly upregulated during both the early and late stages of infection. Functionally, silencing ACSBG1 (siACSBG1) compromised PEDV replication, whereas its overexpression (HA-ACSBG1) enhanced it, corroborating a functional synergy between MCL1 and ACSBG1. We also assessed the contribution of ACSBG1 to the PEDV replication cycle. Interestingly, ACSBG1 overexpression affected PEDV attachment and internalization. Therefore, we speculated that the enhanced PEDV attachment and internalization observed in ACSBG1-overexpressing LLC-PK1 cells were likely due to ACSBG1-induced upregulation of MCL1, since we demonstrated that MCL1 facilitates the early stage of PEDV infection. Moreover, co-overexpression of ACSBG1 and MCL1 markedly enhanced PEDV replication compared with overexpression of ACSBG1 or MCL1 alone, further supporting our idea that ACSBG1 cooperates with MCL1 and primarily functions at the replication step of the PEDV life cycle. A previous study reported that fatty acid β-oxidation supports PEDV replication by providing energy, and that inhibition of β-oxidation markedly impairs viral replication [44]. Consistent with this finding, Etomoxir exhibited pronounced anti-PEDV activity during the replication stage in our study. Moreover, Etomoxir treatment abolished the proviral effects of MCL1 and ACSBG1, either alone or in combination, further supporting the idea that MCL1 cooperates with ACSBG1 to promote PEDV replication through the regulation of cellular β-oxidation.

**Fig 8:**
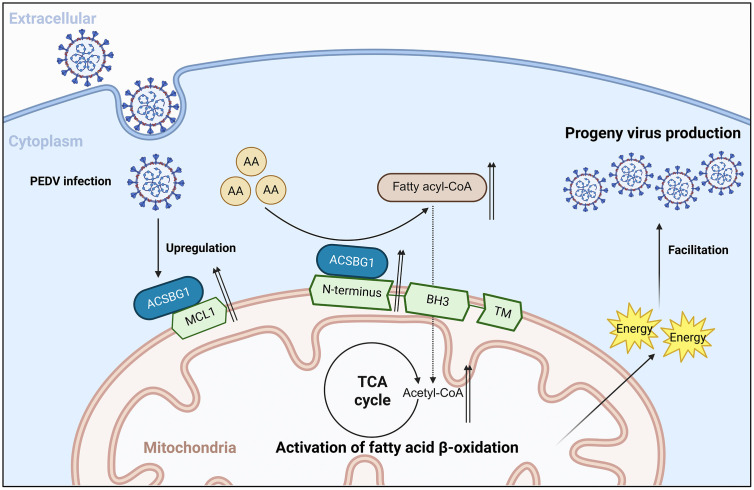
MCL1 cooperates with ACSBG1 to promote PEDV replication by regulating β-oxidation of AA. PEDV infection upregulates MCL1 and ACSBG1. ACSBG1 promotes the conversion of AA into fatty acyl-CoA and, through interaction with the N terminus of MCL1 in a BH domain-dependent manner, further drives the conversion of fatty acyl-CoA into acetyl-CoA, thereby facilitating AA β-oxidation. The resulting acetyl-CoA enters the tricarboxylic acid (TCA) cycle to generate energy, promoting the assembly and production of progeny PEDV. Fig 8 was created using Biorender (Biorender.com; publication and licensing rights agreement TC29IGVL0I). Created in BioRender. niu, N. (2026) https://BioRender.com/kpedsjn.

In conclusion, our data demonstrate that ACSBG1 interacts with the N-terminus of MCL1 to cooperatively activate the AA metabolic pathway. Specifically, the BH3 domain of MCL1 facilitates mitochondrial β-oxidation of AA, thereby generating the energy essential for PEDV replication (Fig 8).

## 4. Materials and methods

### 4.1 Antibodies and reagents

MCL1 polyclonal antibody (pAb) (16225–1-AP), ACSBG1 pAb (16077–1-AP), LC3 pAb (14600–1-AP), PPARA monoclonal antibody (mAb) (66826–1-Ig), PPARD mAb (60193–1-Ig), PPAR Gamma pAb (16643–1-AP), CLTC pAb (26523–1-AP), Caveolin-1 pAb (16447–1-AP), EEA1 pAb (28347–1-AP), RAB7A pAb (55469–1-AP), CD107a/ LAMP1 pAb (21997–1-AP), Beta Actin mAb (66009–1-lg), 6*His, His-tag mAb (66005–1-Ig), 6*His, His-tag pAb (10001–0-AP), HA tag mAb (66006–2-Ig), HA tag pAb (51064–2-AP), horseradish peroxidase (HRP)-conjugated Goat anti-Mouse IgG (H + L) (SA00001–1) and anti-Rabbit (SA00001–2) IgG antibodies, were obtained from Proteintech Group (Wuhan, China). NF-κB p65 antibody (G23D18) and Phospho-NF-κB p65 (Ser536) antibody (M4C8) were obtained from Selleck Incorporation (Houston, USA). PLA2G5 pAb (PC19624) was purchased from Abmart Medical Technology (Shanghai, China). CPT1A pAb (A5307), SQSTM1/p62 mAb (A19700), and GPX4 mAb (A11243), were acquired from ABclonal Technology (Wuhan, China). Caspase-3 pAb (BD-PT0656) was acquired from Biodragon Technology (Beijing, China). In addition, anti-PEDV N monoclonal antibody was prepared in our laboratory. S63845 (HY-100741), arachidonic acid (AA; HY-109590), TK05 (HY-117143), 3-Methyladenine (3-MA; HY-19312), MG132 (HY-13259), Ferrostatin-1 (Fer-1; HY-100579), and Etomoxir (HY-50202) were purchased from MedChemExpress (MCE, NJ, USA). Z-VAD-FMK (C1202) and 4’, 6-diamidino-2-phenylindole (DAPI; C1002) from Beyotime Biotechnology (Shanghai, China). FITC conjugated AffiniPure Goat anti-Mouse IgG (H + L) (BA1101) and CY3 conjugated AffiniPure Goat anti-Rabbit IgG (H + L) (BA1032) was purchased from BOSTER (Wuhan, China). Moreover, the design and synthesis of siRNAs in this study were done by GenePharma (Shanghai, China), and the sequences are listed in S1 Table.

### 4.2 Cell culture

Human hepatocellular carcinoma (Huh7) cells, human embryonic kidney 293T cells (HEK 293T cells; ATCC CRL-11268), porcine kidney cells (LLC-PK1 cells; ATCC CL-101), and swine testis cells (ST cells; ATCC CRL-1746), were cultured in Dulbecco minimal essential medium (DMEM; Gibco, Life Technologies, USA) with 10% heat-inactivated fetal bovine serum (FBS; Biological Industries, Israel) and 1% penicillin-streptomycin (Sigma-Aldrich, USA). Porcine intestinal epithelial (IPEC-J2) cells were maintained in DMEM/F12 medium (Gibco, Life Technologies, USA) supplemented with 10% FBS and 1% penicillin-streptomycin. All the cell lines mentioned above were cultured in humidified 5% CO_2_ and 37°C incubators.

### 4.3 Virus, virus titration, and virus infections

The PEDV strain AH2012/12 (GenBank: KU646831) was isolated and preserved in our laboratory. For viral infection, Huh7 or LLC-PK1 cells were grown to over 90% adherence in culture plates. The cell monolayers were then washed with PBS and inoculated with PEDV at a 0.1 MOI in the presence of 7.5 µg/mL trypsin (Sigma-Aldrich, USA). When obvious cytopathic effects (CPE) were observed, the infected cell cultures were harvested and stored at -80°C. The Reed-Muench method was adopted for determining viral titers of the infected cell cultures, which was shown to reach 50% of tissue culture infective dose TCID_50_ [78].

### 4.4 Plasmid construction

The eukaryotic expression vector pcDNA3.1 (+) was purchased from Sangon Biotech (Shanghai, China) and used as the backbone for all constructs. Plasmids expressing porcine-derived MCL1 and ACSBG1 were first constructed in the laboratory. To investigate the function of the MCL1 domain, we divided MCL1 into three functional domains and constructed their truncated expression vectors, namely N-terminal domain (MCL1_1–175_), the BH domain (MCL1_176–319_), and the transmembrane domain (MCL1_320–351_). The DNA fragments encoding amino acid residues 1–175, 176–319, and 320–351 were amplified from the full-length MCL1 plasmid using it as a template. These amplicons were digested with the restriction enzymes including KpnI and EcoRI (Thermo Fisher Scientific, USA) and subsequently ligated into the pcDNA3.1 (+) vector using T4 DNA Ligase (ABclonal, China). The integrity of all constructed plasmids was verified by DNA sequencing. All primers used for cloning are listed in S2 Table.

### 4.5 Generation of MCL1-KO cell line

To delete MCL1 in LLC-PK1 cells, we employed the CRISPR/Cas9 system. Cells were transiently transfected with pX459 plasmids carrying MCL1 specific sgRNA and Cas9 nuclease. Three distinct sgRNAs targeting sequences upstream of an NGG protospacer adjacent motif (PAM) were designed using a web-based tool provided by the Zhang Lab (https://zlab.bio/guide-design-resources) [79]. These plasmids were generated by ligating synthesized oligonucleotide pairs into the BbsI (New England Biolabs, USA)-linearized pX459 vector. After 36 h of transfection, the cells were selected using puromycin at a dose of 2.0 μg/ml (Beyotime, China) for 48 hours. Selected cells were counted, cloned by limiting dilution, and finally grown in 96-well plates. About 14 days later, the grown-up single clones were collected and were subjected to genomic DNA (gDNA) sequencing and western blot identification. All sgRNA and gDNA validation primer sequences are provided in S3 Table.

### 4.6 Cell viability

The cell viability assays were conducted using a commercial Cell Counting Kit-8 (CCK-8 kits; Beyotime, China) according to the manufacturer instruction to determine the effects of S63845, AA, and TK05 on LLC-PK1 cell viability and proliferation, respectively. The optical density (OD) at 450 nm was measured using a microplate reader (BioTek, USA). Results were expressed relative to the control cells, defined as 100% viability.

### 4.7 Western blot

Cells were lysed on ice with RIPA buffer (Solarbio, China) containing phenylmethanesulfonyl fluoride (PMSF; Solarbio, China). The lysates were clarified by centrifugation and denatured by heating at 95°C for 10 min in sodium dodecyl sulfate-polyacrylamide gel electrophoresis (SDS-PAGE) loading buffer (Beyotime, China). Proteins were then separated by SDS-PAGE and transferred onto 0.22 μm polyvinylidene fluoride membranes (PVDF; Millipore, USA). The membranes were blocked with 5% defatted milk powder dissolved in triethanolamine buffered saline tween buffer (TBST) and subsequently incubated with primary antibodies, followed by incubation with the corresponding HRP-conjugated secondary antibodies. Membranes were washed with TBST between antibody incubations. Protein bands were visualized using an Enhanced Chemiluminescence (ECL) Super Kit (ABclonal, China) and imaged with a Tanon 5200 CE Chemi-Image System (Tanon, China). Band intensities were quantified using ImageJ software, and target protein levels were normalized to ACTB as internal controls.

### 4.8 qRT-PCR

Total RNA was extracted from treated cells using the FastPure Cell/Tissue Total RNA Isolation Kit (Vazyme, China) and subsequently reverse-transcribed into cDNA with the HiScript II Q RT SuperMix (Vazyme, China), following the manufacturer’s protocols. qRT-PCR was then performed using ChamQ Universal SYBR qPCR Master Mix (Vazyme, China) on a QuantStudio 6 Flex system (Applied Biosystems, USA). The thermal cycling conditions were as follows: an initial denaturation at 95˚C for 30 s, followed by 40 cycles of 95˚C for 10 s and 60˚C for 30 s. A melting curve analysis was conducted to ensure product specificity. The relative expression levels of target genes were calculated using 2^−∆∆CT^ method and normalized to ACTB as internal controls. All primer sequences used for qRT-PCR are listed in S4 Table.

### 4.9 RNA-seq

Total RNA was extracted from two million WT and MCL1-KO LLC-PK1 cells. Following quality and integrity assessment, RNA sequencing libraries were constructed and sequenced on an Illumina platform. The reads were mapped to the Sus scrofa reference genome (Ensembl, Release 112). Transcript assembly and differential expression analysis were performed using the Cufflinks suite. Gene expression levels were quantified and normalized as Fragments Per Kilobase of transcript per Million mapped reads (FPKM). DEGs were identified using the criteria of a FDR < 0.05 and an absolute log_2_ (Fold Change) ≥ 2. GO and KEGG pathway enrichment analyses were subsequently performed on the DEGs, and the results were visualized as heatmaps.

### 4.10 AA extraction and quantification

For AA extraction, cells from each group were sequentially treated with 0.25 mL of MS-grade methanol and 0.3 mL of water. The mixture was sonicated for 3 min and then centrifuged at 5000 rpm for 5 min at 4°C. Phase separation was subsequently induced by adding 1 mL of MS-grade methyl-tert-butyl ether (MTBE). The upper organic phase was collected, and the lower aqueous phase was re-extracted with 1 mL of a solvent mixture (MTBE/methanol/water, 10:3:2.5, v/v/v). The upper phase from the re-extraction was collected and pooled with first extraction. The combined organic phases were dried under nitrogen, and the residue was reconstituted in 500 μL of MS-grade methanol.

For AA quantification, standard solutions were first obtained by serial dilution of AA using MS grade methanol at concentrations from 10 to 2000 ng/mL. The calibration curve was then determined to include 6–9 calibration points. Each sample was filtered through a 0.22 µm membrane and transferred to a glass tube with a Teflon lined cap, vortexed and analyzed with an HPLC-MS/MS system (Agilent Technology, USA). After subtraction of the blank, the AA contents of the samples were quantified by using linear regression from the calibration curve (ratio of peak area analyte vs. analyte concentration).

### 4.11 Acetyl-CoA assay

The intracellular acetyl-CoA levels in WT and MCL1-KO LLC-PK1 cells were detected using a commercial assay kit (Solarbio, China) according to the manufacturer’s protocol. Briefly, cell pellets containing 5 × 10^6^ cells were lysed with a mixture of 0.99 mL of Extract I and 0.01 mL of Extract II. The lysates were sonicated on ice and then clarified by centrifugation at 12000 rpm for 10 min at 4 °C. Subsequently, 200 μL of the supernatant was mixed with 820 μL of a freshly prepared working solution. The absorbance at 340 nm was immediately measured at 20 s and 80 s using an using an ultraviolet spectrophotometer (Agilent Technology, USA).

### 4.12 Confocal immunofluorescence assay

The treated cells were fixed in pre-chilled absolute ethanol for 30 min, followed by permeabilization with 0.1% (v/v) Triton X-100 in PBS for 10 min. After blocking with 5% bovine serum albumin (BSA; Solarbio, China), cells were sequentially incubated with indicated primary antibodies for 2 h, followed by incubation with the corresponding fluorophore-conjugated secondary antibodies for 1 h. Nuclei were counterstained with DAPI (Beyotime, China) for 5 min. All incubations were performed at room temperature, and cells were washed thoroughly with PBS between each step. Fluorescence images were acquired using a Zeiss LSM880 confocal microscope (Carl Zeiss AG, Germany).

### 4.13 Co-IP assay

HEK293T cells were transfected with the indicated plasmids for 36 h. The cells were then lysed in IP lysis buffer (Beyotime, China) on ice for 15 min. Cell debris was removed by centrifugation at 12000 rpm for 10 min at 4 °C. A small aliquot (10%) of the supernatant was reserved as the input control. The remaining lysate was incubated with the appropriate primary antibody (anti-HA or anti-His) overnight, followed by incubation with 40 μL of Protein A + G Agarose beads (Beyotime, China) for 2 h. Both incubations were conducted at 4 °C with constant rotation. The beads were subsequently washed extensively with PBS and the immunoprecipitated proteins were eluted by boiling in SDS-PAGE loading buffer for 10 min. The immunoprecipitates and the corresponding input controls were analyzed via western blot analysis using the indicated antibodies.

### 4.14 Computational modeling and structural analysis of interacting proteins

To investigate potential molecular interactions, we employed computational modeling to predict the structure of the protein complex using the AlphaFold2-Multimer pipeline with their canonical amino acid sequences as input. The pipeline generated five structural models, which were ranked based on confidence scores. The reliability of these predictions was evaluated by three key metrics: the per-residue confidence score (pLDDT), the interface predicted Template Modeling (ipTM) score, and the Predicted Aligned Error (PAE) plot. Ultimately, the model exhibiting the highest ipTM score coupled with the most favorable PAE profile at the protein-protein interface was chosen as the most plausible structural representation. This final model was subsequently visualized and analyzed in PyMOL (v2.5).

### 4.15 Statistical analysis

Data from three independent experiments were used to calculate mean ± standard deviation (SD). Statistical analyses were performed using SPSS 26. For multi-group comparisons, a one-way analysis of variance (ANOVA) followed by a post-hoc Least Significant Difference (LSD) test was applied. For two-group comparisons, a two-tailed Student’s t-test was used. *P*-values < 0.05 were considered statistically significant, with specific levels denoted as *P < 0.05, **P < 0.01, and ***P < 0.001; ‘ns’ indicates non-significance. All figures were generated in GraphPad Prism 8 (GraphPad Software).

## Supporting information

S1 Fig Validation of CRISPR-Cas9 knockout screening in MCL1-KO Huh7 cells and selection of porcine cell model for MCL1 study. (A to D) The effect of MCL1-KO in Huh7 cells on PEDV infection. The MCL1-KO and WT Huh7 cells were infected with PEDV (AH2012/12, MOI = 1) for 24 h. The cell samples were harvested and PEDV N mRNA expression was detected by qRT-PCR (A), PEDV N and MCL1 protein levels were detected by western blot (B). Quantitative comparisons of PEDV N were analyzed by gray intensity scanning of blots (C). Cell culture supernatants were collected and virus levels were determined using TCID_50_ assays (D). (E to G) The mRNA and protein expression of MCL1 in different swine somatic cells including IPEC J2 cells, LLC-PK1 cells, and ST cells, were detected by qRT-PCR (E) and western blot (F). The band intensity of MCL1 was quantified using ImageJ software (G). The presented results represent the means and standard deviations of the data from three independent experiments. *, *P* < 0.05; **, *P* < 0.01.
(TIF)

S2 Fig PCD is not involved in the MCL1-mediated PEDV infection. (A) MCL1-KO and WT LLC-PK1 cells were infected with PEDV (AH2012/12, MOI = 0.1) for 16 h, and then the cells were treated with Z-VAD-FMK (20 μM), Fer-1 (10 μM), and 3-MA (5 mM) respectively for 8 h. The protein expression levels of PEDV N, caspase-3, GPX4, LC3, and ACTB were detected by western blot. (B) The band intensity of PEDV N from each group was quantified using ImageJ software. ***, *P* < 0.001;
(TIF)

S3 Fig KEGG and GO enrichment analysis of DEGs. The PPAR signaling pathway (A) and the fat digestion and absorption pathway (B) derived from the KEGG database were shown. The upregulated DEGs enriched in the two pathways were enclosed in red boxes. (C) GO enrichment bar chart derived from the transcriptomic results was shown.
(TIF)

S4 Fig Screening of ACS family member regulated MCL1 and interaction sites with MCL1. ACSL4 mRNA relative expression and protein levels in MCL1-KO and WT LLC-PK1 cells were measured by qRT-PCR (A) and western blot (B). The band intensity of ACSL4 was quantified using ImageJ software (C). DEGs in the ACS family identified from the transcriptomic dataset are shown (D). ACSBG1-HA were immunoprecipitated with anti-His binding beads (E), and MCL1_176–319_-His were immunoprecipitated with anti-HA binding beads (F). HEK293T cells transfected with corresponding empty vectors served as the controls. Ribbon diagram represented the structure of human-derived ACSBG1-MCL1 complex with ACSBG1 colored in blue and MCL1 in red (G). Close-up views of the interface highlighted specific residues from ACSBG1 (blue) and MCL1 (red), and the predicted interaction sites were indicated by yellow bonds (H-J). The presented results represent the means and standard deviations of the data from three independent experiments. Ns, not significant.
(TIF)

S1 Table The sequences of siRNAs used in this study. (DOCX)

S2 Table The primers used for cloning in this study. (DOCX)

S3 Table The sequences of the sgRNAs and primers used to generate and validate the MCL1-KO cell lines. (DOCX)

S4 Table The primers used for qRT-PCR in this study. (DOCX)

## References

[1] Wang D, Fang L, Xiao S. Porcine epidemic diarrhea in China. Virus Res. 2016;226:7–13. doi: 10.1016/j.virusres.2016.05.026 27261169 PMC7114554

[2] Jung K, Saif LJ, Wang Q. Porcine epidemic diarrhea virus (PEDV): an update on etiology, transmission, pathogenesis, and prevention and control. Virus Res. 2020;286:198045. doi: 10.1016/j.virusres.2020.198045 32502552 PMC7266596

[3] Pensaert MB, de Bouck P. A new coronavirus-like particle associated with diarrhea in swine. Arch Virol. 1978;58(3):243–7. doi: 10.1007/BF01317606 83132 PMC7086830

[4] Takahashi K, Okada K, Ohshima K. An outbreak of swine diarrhea of a new-type associated with coronavirus-like particles in Japan. Nihon Juigaku Zasshi. 1983;45(6):829–32. doi: 10.1292/jvms1939.45.829 6323804

[5] Stevenson GW, Hoang H, Schwartz KJ, Burrough ER, Sun D, Madson D, et al. Emergence of Porcine epidemic diarrhea virus in the United States: clinical signs, lesions, and viral genomic sequences. J Vet Diagn Invest. 2013;25(5):649–54. doi: 10.1177/1040638713501675 23963154

[6] Chen Q, Li G, Stasko J, Thomas JT, Stensland WR, Pillatzki AE, et al. Isolation and characterization of porcine epidemic diarrhea viruses associated with the 2013 disease outbreak among swine in the United States. J Clin Microbiol. 2014;52(1):234–43. doi: 10.1128/JCM.02820-13 24197882 PMC3911415

[7] Mole B. Deadly pig virus slips through US borders. Nature. 2013;499(7459):388. doi: 10.1038/499388a 23887408

[8] Guo J, Fang L, Ye X, Chen J, Xu S, Zhu X, et al. Evolutionary and genotypic analyses of global porcine epidemic diarrhea virus strains. Transbound Emerg Dis. 2019;66(1):111–8. doi: 10.1111/tbed.12991 30102851 PMC7168555

[9] Li W, Li H, Liu Y, Pan Y, Deng F, Song Y, et al. New variants of porcine epidemic diarrhea virus, China, 2011. Emerg Infect Dis. 2012;18(8):1350–3. doi: 10.3201/eid1808.120002 22840964 PMC3414035

[10] Wei M-Z, Chen L, Zhang R, Chen Z, Shen Y-J, Zhou B-J, et al. Overview of the recent advances in porcine epidemic diarrhea vaccines. Vet J. 2024;304:106097. doi: 10.1016/j.tvjl.2024.106097 38479492

[11] Zhao Y, Fan B, Song X, Gao J, Guo R, Yi C, et al. PEDV-spike-protein-expressing mRNA vaccine protects piglets against PEDV challenge. mBio. 2024;15(2):e0295823. doi: 10.1128/mbio.02958-23 38231557 PMC10865985

[12] Zhang D, Xie Y, Liao Q, Jiao Z, Liang R, Zhang J, et al. Development of a safe and broad-spectrum attenuated PEDV vaccine candidate by S2 subunit replacement. J Virol. 2024;98(11):e0042924. doi: 10.1128/jvi.00429-24 39404450 PMC11575183

[13] Wang J, Liu H, Yang Y, Tan Y, Sun L, Guo Z, et al. Genome-scale CRISPR screen identifies TRIM2 and SLC35A1 associated with porcine epidemic diarrhoea virus infection. Int J Biol Macromol. 2023;250:125962. doi: 10.1016/j.ijbiomac.2023.125962 37499712

[14] Zhou J, Feng Z, Lv D, Wang D, Sang K, Liu Z, et al. Unveiling the role of protein kinase C θ in porcine epidemic diarrhea virus replication: insights from genome-wide CRISPR/Cas9 library screening. Int J Mol Sci. 2024;25(6):3096. doi: 10.3390/ijms25063096 38542067 PMC10969977

[15] Lv L, Luo H, Yi J, Zhang K, Li Y, Tong W, et al. IFITM proteins are key entry factors for porcine epidemic diarrhea coronavirus. J Virol. 2025;99(6):e0202824. doi: 10.1128/jvi.02028-24 40353666 PMC12172462

[16] Guo L, Duan X, Li J, Hao Z, Bi Y, Chen Y, et al. YIPF5 is an essential host factor for porcine epidemic diarrhea virus double-membrane vesicle formation. J Virol. 2025;99(6):e0032025. doi: 10.1128/jvi.00320-25 40422075 PMC12172457

[17] Guo G, Zhang M, Xu Z, Xi P, Zhu H, Evers A, et al. Genome-wide CRISPR screen reveals key role of sialic acids in PEDV and porcine coronavirus infections. mBio. 2025;16(9):e0162825. doi: 10.1128/mbio.01628-25 40767522 PMC12421841

[18] Zhao Y, Guo G, Sun Y, Zhang M, Yang G, Liu Z, et al. Membrane protein CRISPR screen identifies RPSA as an essential host factor for porcine epidemic diarrhea virus replication. J Virol. 2025;99(8):e0064925. doi: 10.1128/jvi.00649-25 40736249 PMC12363229

[19] Kozopas KM, Yang T, Buchan HL, Zhou P, Craig RW. MCL1, a gene expressed in programmed myeloid cell differentiation, has sequence similarity to BCL2. Proc Natl Acad Sci U S A. 1993;90(8):3516–20. doi: 10.1073/pnas.90.8.3516 7682708 PMC46331

[20] Denis C, Sopková-de Oliveira Santos J, Bureau R, Voisin-Chiret AS. Hot-Spots of Mcl-1 protein: miniperspective. J Med Chem. 2020;63:928–43. doi: 10.1021/acs.jmedchem.9b00983 31580668

[21] Senichkin VV, Streletskaia AY, Zhivotovsky B, Kopeina GS. Molecular comprehension of Mcl-1: from gene structure to cancer therapy. Trends Cell Biol. 2019;29(7):549–62. doi: 10.1016/j.tcb.2019.03.004 31030977

[22] Widden H, Placzek WJ. The multiple mechanisms of MCL1 in the regulation of cell fate. Commun Biol. 2021;4(1):1029. doi: 10.1038/s42003-021-02564-6 34475520 PMC8413315

[23] Wyżewski Z, Stępkowska J, Kobylińska AM, Mielcarska A, Mielcarska MB. Mcl-1 protein and viral infections: a narrative review. Int J Mol Sci. 2024;25(2):1138. doi: 10.3390/ijms25021138 38256213 PMC10816053

[24] Zhang X, Fan B, Zhao Y, Qian J, Wang C, Xu H. Screening and validation of porcine epidemic diarrhea virus replication-related genes based on genome-scale CRISPR/Cas9 system. Microbiology China. 2022;49:5138–49. doi: 10.13344/j.microbiol.china.220895

[25] Fan B, Zhu L, Chang X, Zhou J, Guo R, Zhao Y, et al. Mortalin restricts porcine epidemic diarrhea virus entry by downregulating clathrin-mediated endocytosis. Vet Microbiol. 2019;239:108455. doi: 10.1016/j.vetmic.2019.108455 31767073

[26] Zhou C, Liu Y, Wei Q, Chen Y, Yang S, Cheng A, et al. HSPA5 promotes attachment and internalization of porcine epidemic diarrhea virus through interaction with the spike protein and the endo-/lysosomal pathway. J Virol. 2023;97(6):e0054923. doi: 10.1128/jvi.00549-23 37222617 PMC10308931

[27] Mao B, Le-Trilling VTK, Wang K, Mennerich D, Hu J, Zhao Z, et al. Obatoclax inhibits SARS-CoV-2 entry by altered endosomal acidification and impaired cathepsin and furin activity in vitro. Emerg Microbes Infect. 2022;11(1):483–97. doi: 10.1080/22221751.2022.2026739 34989664 PMC8843317

[28] Wei X, She G, Wu T, Xue C, Cao Y. PEDV enters cells through clathrin-, caveolae-, and lipid raft-mediated endocytosis and traffics via the endo-/lysosome pathway. Vet Res. 2020;51. doi: 10.1186/s13567-020-0739-7 PMC7011528 32041637

[29] Peng Q, Fan B, Song X, He W, Wang C, Zhao Y, et al. Genetic signatures associated with the virulence of porcine epidemic diarrhea virus AH2012/12. J Virol. 2023;97(10):e0106323. doi: 10.1128/jvi.01063-23 37732788 PMC10617547

[30] Fu Y, Fu Z, Su Z, Li L, Yang Y, Tan Y, et al. mLST8 is essential for coronavirus replication and regulates its replication through the mTORC1 pathway. mBio. 2023;14(4):e0089923. doi: 10.1128/mbio.00899-23 37377422 PMC10470783

[31] Kotschy A, Szlavik Z, Murray J, Davidson J, Maragno AL, Le Toumelin-Braizat G, et al. The MCL1 inhibitor S63845 is tolerable and effective in diverse cancer models. Nature. 2016;538(7626):477–82. doi: 10.1038/nature19830 27760111

[32] Petros AM, Olejniczak ET, Fesik SW. Structural biology of the Bcl-2 family of proteins. Biochim Biophys Acta - Mol Cell Res. 2004;1644(2–3):83–94. doi: 10.1016/j.bbamcr.2003.08.012 14996493

[33] Chong SJF, Marchi S, Petroni G, Kroemer G, Galluzzi L, Pervaiz S. Noncanonical cell fate regulation by Bcl-2 proteins. Trends Cell Biol. 2020;30:537–55. doi: 10.1016/j.tcb.2020.03.004 32307222

[34] Aghasizadeh M, Bahrami AR, Matin MM. Polyunsaturated fatty acids in kidney diseases: navigating the fine line between healing and damage. Biochim Biophys Acta Mol Cell Biol Lipids. 2025;1870(7):159668. doi: 10.1016/j.bbalip.2025.159668 40712926

[35] Wang T, Fu X, Chen Q, Patra JK, Wang D, Wang Z, et al. Arachidonic acid metabolism and kidney inflammation. Int J Mol Sci. 2019;20(15):3683. doi: 10.3390/ijms20153683 31357612 PMC6695795

[36] Baba T, Black KL, Ikezaki K, Chen KN, Becker DP. Intracarotid infusion of leukotriene C4 selectively increases blood-brain barrier permeability after focal ischemia in rats. J Cereb Blood Flow Metab. 1991;11(4):638–43. doi: 10.1038/jcbfm.1991.115 1675639

[37] Balestrieri B, Arm JP. Group V sPLA2: classical and novel functions. Biochim Biophys Acta Mol Cell Biol Lipids. 2006;1761:1280–8. doi: 10.1016/j.bbalip.2006.07.008 16945583

[38] Kleinschmidt TK, Haraldsson M, Basavarajappa D, Lundeberg E, Thulasingam M, Ekoff M, et al. Tandem benzophenone amino pyridines, potent and selective inhibitors of human leukotriene C4 synthase. J Pharmacol Exp Ther. 2015;355(1):108–16. doi: 10.1124/jpet.115.227157 26283693

[39] Escudero S, Zaganjor E, Lee S, Mill CP, Morgan AM, Crawford EB, et al. Dynamic regulation of long-chain fatty acid oxidation by a noncanonical interaction between the MCL-1 BH3 helix and VLCAD. Mol Cell. 2018;69(5):729-743.e7. doi: 10.1016/j.molcel.2018.02.005 29499131 PMC5916823

[40] Bhargava P, Schnellmann RG. Mitochondrial energetics in the kidney. Nat Rev Nephrol. 2017;13(10):629–46. doi: 10.1038/nrneph.2017.107 28804120 PMC5965678

[41] Tomitsuka Y, Imaeda H, Ito H, Asou I, Ohbayashi M, Ishikawa F, et al. Gene deletion of long-chain acyl-CoA synthetase 4 attenuates xenobiotic chemical-induced lung injury via the suppression of lipid peroxidation. Redox Biol. 2023;66:102850. doi: 10.1016/j.redox.2023.102850 37586249 PMC10450978

[42] Mishra S, Shelke V, Gaikwad AB. Acyl-CoA synthetase long-chain isoenzymes in kidney diseases: mechanistic insights and therapeutic implications. Cell Biochem Funct. 2024;42(7):e4114. doi: 10.1002/cbf.4114 39210707

[43] Wright T, Turnis ME, Grace CR, Li X, Brakefield LA, Wang Y-D, et al. Anti-apoptotic MCL-1 promotes long-chain fatty acid oxidation through interaction with ACSL1. Mol Cell. 2024;84(7):1338-1353.e8. doi: 10.1016/j.molcel.2024.02.035 38503284 PMC11017322

[44] Wang Q, Zhang Q, Shi X, Yang N, Zhang Y, Li S, et al. ACADM inhibits AMPK activation to modulate PEDV-induced lipophagy and β-oxidation for impairing viral replication. J Biol Chem. 2024;300(8):107549. doi: 10.1016/j.jbc.2024.107549 39002673 PMC11342783

[45] Raud B, Roy DG, Divakaruni AS, Tarasenko TN, Franke R, Ma EH, et al. Etomoxir actions on regulatory and memory T cells are independent of Cpt1a-mediated fatty acid oxidation. Cell Metab. 2018;28(3):504-515.e7. doi: 10.1016/j.cmet.2018.06.002 30043753 PMC6747686

[46] Chunhacha P, Pongrakhananon V, Rojanasakul Y, Chanvorachote P. Caveolin-1 regulates Mcl-1 stability and anoikis in lung carcinoma cells. Am J Physiol Cell Physiol. 2012;302(9):C1284-92. doi: 10.1152/ajpcell.00318.2011 22277751 PMC3774262

[47] Pan P, Ge W, Lei Z, Luo W, Liu Y, Guan Z, et al. SARS-CoV-2 N protein enhances the anti-apoptotic activity of MCL-1 to promote viral replication. Signal Transduct Target Ther. 2023;8(1):194. doi: 10.1038/s41392-023-01459-8 37160897 PMC10169150

[48] Perciavalle RM, Stewart DP, Koss B, Lynch J, Milasta S, Bathina M, et al. Anti-apoptotic MCL-1 localizes to the mitochondrial matrix and couples mitochondrial fusion to respiration. Nat Cell Biol. 2012;14:575–83. doi: 10.1038/ncb2488 22544066 PMC3401947

[49] Kumar R, Mehta D, Mishra N, Nayak D, Sunil S. Role of host-mediated post-translational modifications (PTMs) in RNA virus pathogenesis. Int J Mol Sci. 2020;22(1):323. doi: 10.3390/ijms22010323 33396899 PMC7796338

[50] Mello-Vieira J, Dikic I. Ubiquitination and autophagy in host-pathogen interactions: from immune surveillance to therapeutic targeting. Nat Rev Immunol. 2026. doi: 10.1038/s41577-025-01239-1 41491841

[51] Orzalli MH, Prochera A, Payne L, Smith A, Garlick JA, Kagan JC. Virus-mediated inactivation of anti-apoptotic Bcl-2 family members promotes Gasdermin-E-dependent pyroptosis in barrier epithelial cells. Immunity. 2021;54(7):1447-1462.e5. doi: 10.1016/j.immuni.2021.04.012 33979579 PMC8594743

[52] Guan Z, Li H, Zhang C, Huang Z, Ye M, Zhang Y, et al. RVFV virulence factor NSs triggers the mitochondrial MCL-1-BAK axis to activate pathogenic NLRP3 pyroptosis. PLoS Pathog. 2024;20(8):e1012387. doi: 10.1371/journal.ppat.1012387 39213434 PMC11364418

[53] Brinkmann K, McArthur K, Malelang S, Gibson L, Tee A, Elahee Doomun SN, et al. Relative importance of the anti-apoptotic versus apoptosis-unrelated functions of MCL-1 in vivo. Science. 2025;389(6764):1003–11. doi: 10.1126/science.adw1836 40608895

[54] Pérez-Chacón G, Astudillo AM, Balgoma D, Balboa MA, Balsinde J. Control of free arachidonic acid levels by phospholipases A2 and lysophospholipid acyltransferases. Biochim Biophys Acta. 2009;1791(12):1103–13. doi: 10.1016/j.bbalip.2009.08.007 19715771

[55] Bouhaddou M, Memon D, Meyer B, White KM, Rezelj VV, Correa Marrero M, et al. The global phosphorylation landscape of SARS-CoV-2 infection. Cell. 2020;182(3):685-712.e19. doi: 10.1016/j.cell.2020.06.034 32645325 PMC7321036

[56] Ravindra NG, Alfajaro MM, Gasque V, Huston NC, Wan H, Szigeti-Buck K, et al. Single-cell longitudinal analysis of SARS-CoV-2 infection in human airway epithelium identifies target cells, alterations in gene expression, and cell state changes. PLoS Biol. 2021;19(3):e3001143. doi: 10.1371/journal.pbio.3001143 33730024 PMC8007021

[57] DeDiego ML, Nieto-Torres JL, Regla-Nava JA, Jimenez-Guardeño JM, Fernandez-Delgado R, Fett C, et al. Inhibition of NF-κB-mediated inflammation in severe acute respiratory syndrome coronavirus-infected mice increases survival. J Virol. 2014;88(2):913–24. doi: 10.1128/JVI.02576-13 24198408 PMC3911641

[58] Nilsson-Payant BE, Uhl S, Grimont A, Doane AS, Cohen P, Patel RS, et al. The NF-κB transcriptional footprint is essential for SARS-CoV-2 replication. J Virol. 2021;95(23):e0125721. doi: 10.1128/JVI.01257-21 34523966 PMC8577386

[59] Kandasamy M. NF-κB signalling as a pharmacological target in COVID-19: potential roles for IKKβ inhibitors. Naunyn Schmiedebergs Arch Pharmacol. 2021;394(3):561–7. doi: 10.1007/s00210-020-02035-5 33394134 PMC7780215

[60] Sun P, Wang M, Li J, Qiu Y, Li H, Lv M, et al. Inhibitory effect of Buddlejasaponin IVb on porcine epidemic diarrhea virus in vivo and in vitro. Vet Microbiol. 2022;272:109516. doi: 10.1016/j.vetmic.2022.109516 35901581

[61] Wei Y, Zhao S, Zhao H, Bao D, Liu J, Shao H, et al. FoxO4 reduces the damage and mechanism of PEDV-infected IPEC-J2 cells through the NF-κB/MLCK pathway. Vet Microbiol. 2025;306:110568. doi: 10.1016/j.vetmic.2025.110568 40398348

[62] Wu Y, Wang Y, Wang X, Li M, Yan H, Shi H, et al. Elevation of IL-8 secretion induced by PEDV infection via NF-κB signaling pathway. Front Cell Infect Microbiol. 2024;14:1422560. doi: 10.3389/fcimb.2024.1422560 39104852 PMC11298435

[63] Zhou GL, Beloiartsev A, Yu B, Baron DM, Zhou W, Niedra R, et al. Deletion of the murine cytochrome P450 Cyp2j locus by fused BAC-mediated recombination identifies a role for Cyp2j in the pulmonary vascular response to hypoxia. PLoS Genet. 2013;9(11):e1003950. doi: 10.1371/journal.pgen.1003950 24278032 PMC3836722

[64] Suo X, Wang J, Wang D, Fan G, Zhu M, Fan B, et al. DHA and EPA inhibit porcine coronavirus replication by alleviating ER stress. J Virol. 2023;97(11):e0120923. doi: 10.1128/jvi.01209-23 37843366 PMC10688372

[65] Yang S, Huang X, Li S, Wang C, Jansen CA, Savelkoul HFJ, et al. Linoleic acid: a natural feed compound against porcine epidemic diarrhea disease. J Virol. 2023;97(12):e0170023. doi: 10.1128/jvi.01700-23 38009930 PMC10734519

[66] Miura Y. The biological significance of ω-oxidation of fatty acids. Proc Jpn Acad Ser B Phys Biol Sci. 2013;89(8):370–82. doi: 10.2183/pjab.89.370 24126285 PMC3832743

[67] Prew MS, Adhikary U, Choi DW, Portero EP, Paulo JA, Gowda P, et al. MCL-1 is a master regulator of cancer dependency on fatty acid oxidation. Cell Rep. 2022;41(1):111445. doi: 10.1016/j.celrep.2022.111445 36198266 PMC9933948

[68] Tillander V, Alexson SEH, Cohen DE. Deactivating fatty acids: Acyl-CoA thioesterase-mediated control of lipid metabolism. Trends Endocrinol Metab. 2017;28(7):473–84. doi: 10.1016/j.tem.2017.03.001 28385385 PMC5474144

[69] Kuwata H, Hara S. Role of acyl-CoA synthetase ACSL4 in arachidonic acid metabolism. Prostagl Other Lipid Mediat. 2019;144:106363. doi: 10.1016/j.prostaglandins.2019.106363 31306767

[70] Doll S, Proneth B, Tyurina YY, Panzilius E, Kobayashi S, Ingold I, et al. ACSL4 dictates ferroptosis sensitivity by shaping cellular lipid composition. Nat Chem Biol. 2017;13(1):91–8. doi: 10.1038/nchembio.2239 27842070 PMC5610546

[71] Jiang X, Stockwell BR, Conrad M. Ferroptosis: mechanisms, biology and role in disease. Nat Rev Mol Cell Biol. 2021;22:266–82. doi: 10.1038/s41580-020-00324-8 33495651 PMC8142022

[72] Capdevila JH, Falck JR, Harris RC. Cytochrome P450 and arachidonic acid bioactivation. Molecular and functional properties of the arachidonate monooxygenase. J Lipid Res. 2000;41(2):163–81. doi: 10.1016/s0022-2275(20)32049-6 10681399

[73] Zeldin DC. Epoxygenase pathways of arachidonic acid metabolism. J Biol Chem. 2001;276(39):36059–62. doi: 10.1074/jbc.R100030200 11451964

[74] Peters-Golden M, Henderson WR. Leukotrienes. N Engl J Med. 2007;357:1841–54. doi: 10.1056/NEJMra071371 17978293

[75] Steinberg SJ, Morgenthaler J, Heinzer AK, Smith KD, Watkins PA. Very long-chain Acyl-CoA synthetases. J Biol Chem. 2000;275:35162–9. doi: 10.1074/jbc.M006403200 10954726

[76] Ye X, Li Y, González-Lamuño D, Pei Z, Moser AB, Smith KD, et al. Role of ACSBG1 in brain lipid metabolism and X-linked adrenoleukodystrophy pathogenesis: insights from a knockout mouse model. Cells. 2024;13(20):1687. doi: 10.3390/cells13201687 39451204 PMC11506745

[77] Banjara S, Suraweera CD, Hinds MG, Kvansakul M. The Bcl-2 family: ancient origins, conserved structures, and divergent mechanisms. Biomolecules. 2020;10(1):128. doi: 10.3390/biom10010128 31940915 PMC7022251

[78] Pizzi M. Sampling variation of the fifty percent end-point, determined by the Reed-Muench (Behrens) method. Hum Biol. 1950;22(3):151–90. 14778593

[79] Hsu PD, Scott DA, Weinstein JA, Ran FA, Konermann S, Agarwala V, et al. DNA targeting specificity of RNA-guided Cas9 nucleases. Nat Biotechnol. 2013;31(9):827–32. doi: 10.1038/nbt.2647 23873081 PMC3969858

