# Supplementary figures and images for: MCL1 promotes porcine epidemic diarrhea virus replication by modulating arachidonic acid metabolic pathway
Source: PLoS Pathog. 2026 Apr 24;22(4):e1014170. doi: 10.1371/journal.ppat.1014170 (PMC13138738; doi:10.1371/journal.ppat.1014170)

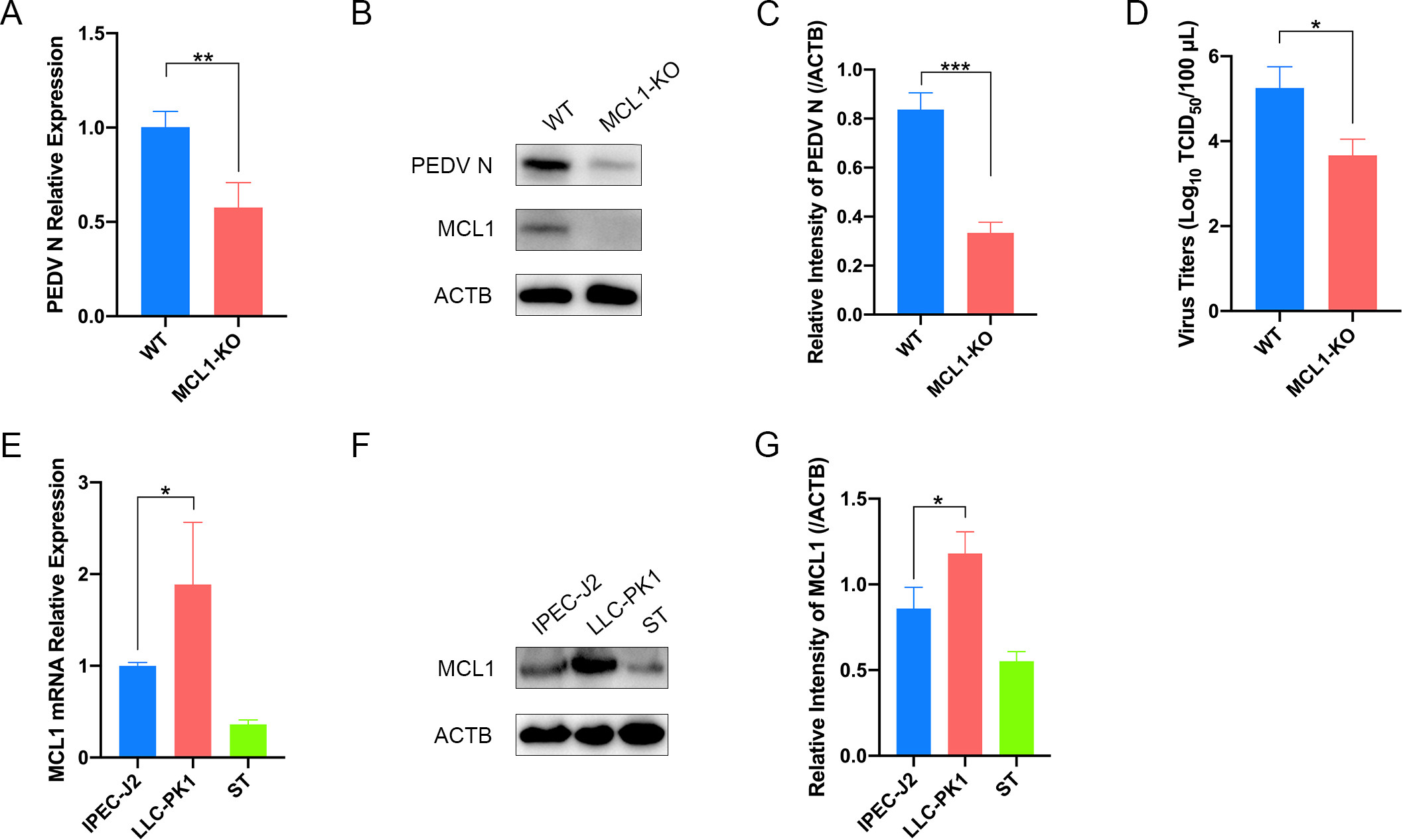

Supplement: S1 Fig — (A to D) The effect of MCL1-KO in Huh7 cells on PEDV infection. The MCL1-KO and WT Huh7 cells were infected with PEDV (AH2012/12, MOI = 1) for 24 h. The cell samples were harvested and PEDV N mRNA expression was detected by qRT-PCR (A), PEDV N and MCL1 protein levels were detected by western blot (B). Quantitative comparisons of PEDV N were analyzed by gray intensity scanning of blots (C). Cell culture supernatants were collected and virus levels were determined using TCID50 assays (D). (E to G) The mRNA and protein expression of MCL1 in different swine somatic cells including IPEC J2 cells, LLC-PK1 cells, and ST cells, were detected by qRT-PCR (E) and western blot (F). The band intensity of MCL1 was quantified using ImageJ software (G). The presented results represent the means and standard deviations of the data from three independent experiments. *, P < 0.05; **, P < 0.01. (TIF) [file ppat.1014170.s001.tif]

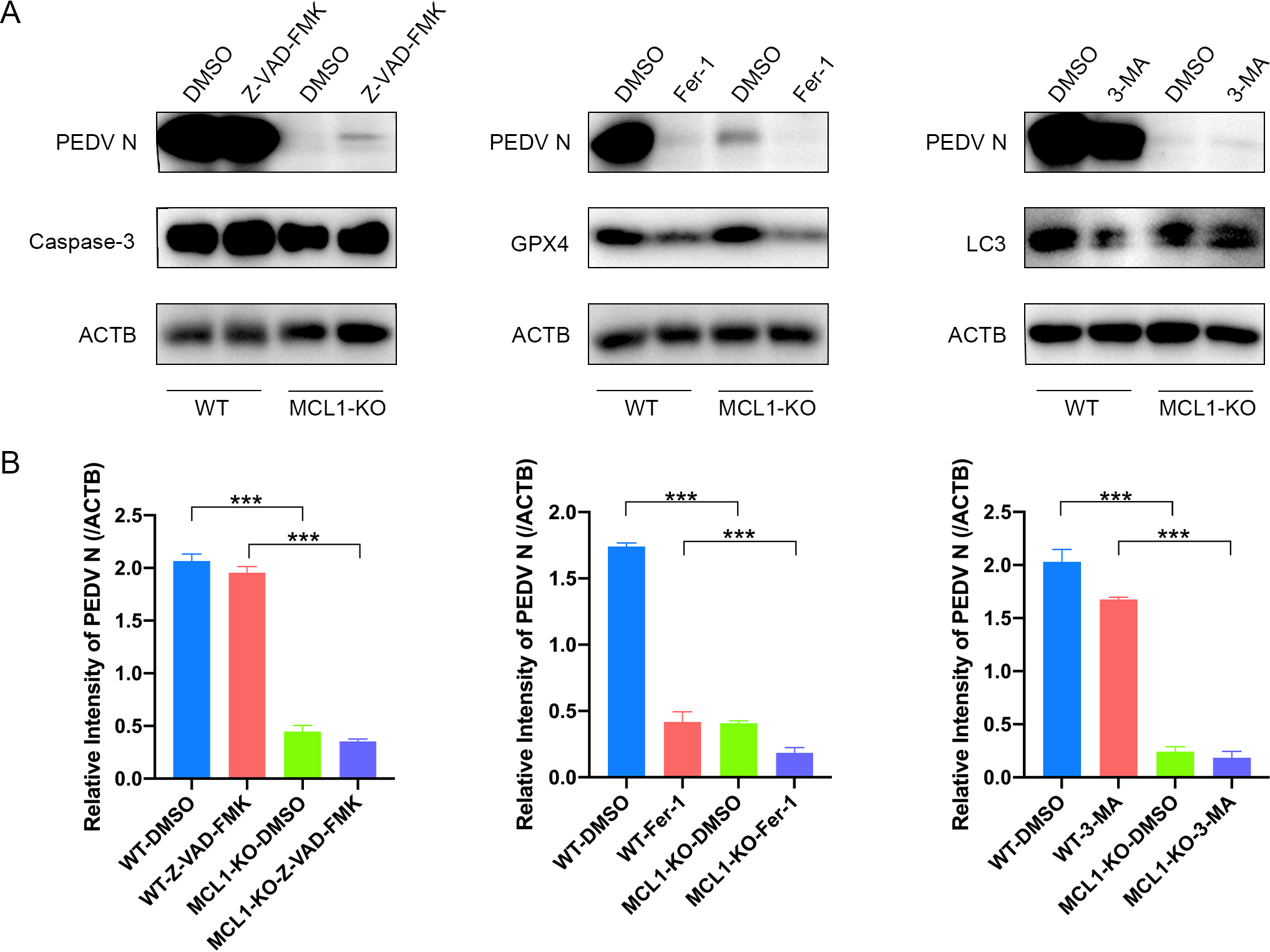

Supplement: S2 Fig — (A) MCL1-KO and WT LLC-PK1 cells were infected with PEDV (AH2012/12, MOI = 0.1) for 16 h, and then the cells were treated with Z-VAD-FMK (20 μM), Fer-1 (10 μM), and 3-MA (5 mM) respectively for 8 h. The protein expression levels of PEDV N, caspase-3, GPX4, LC3, and ACTB were detected by western blot. (B) The band intensity of PEDV N from each group was quantified using ImageJ software. ***, P < 0.001; (TIF) [file ppat.1014170.s002.tif]

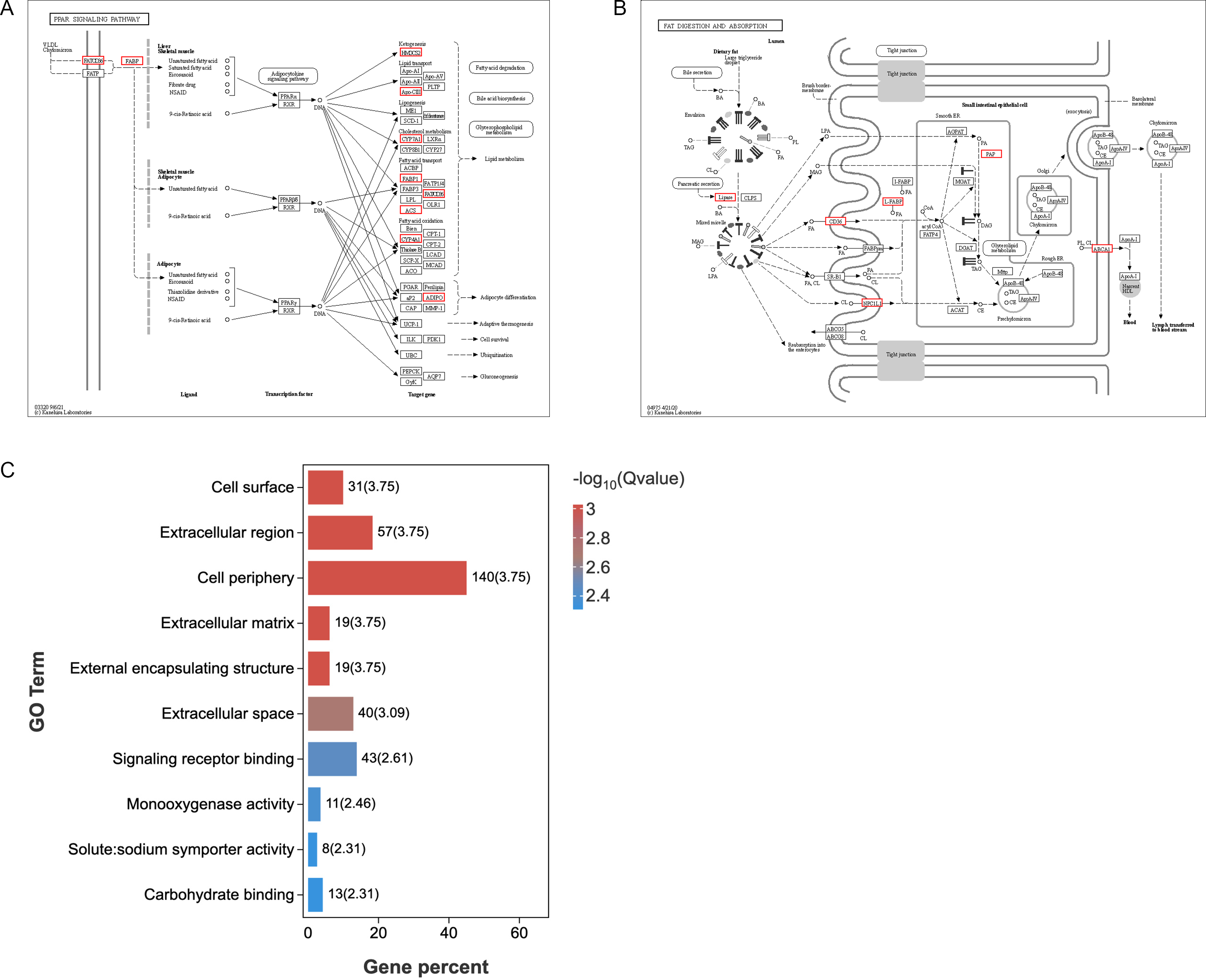

Supplement: S3 Fig — The PPAR signaling pathway (A) and the fat digestion and absorption pathway (B) derived from the KEGG database were shown. The upregulated DEGs enriched in the two pathways were enclosed in red boxes. (C) GO enrichment bar chart derived from the transcriptomic results was shown. (TIF) [file ppat.1014170.s003.tif]

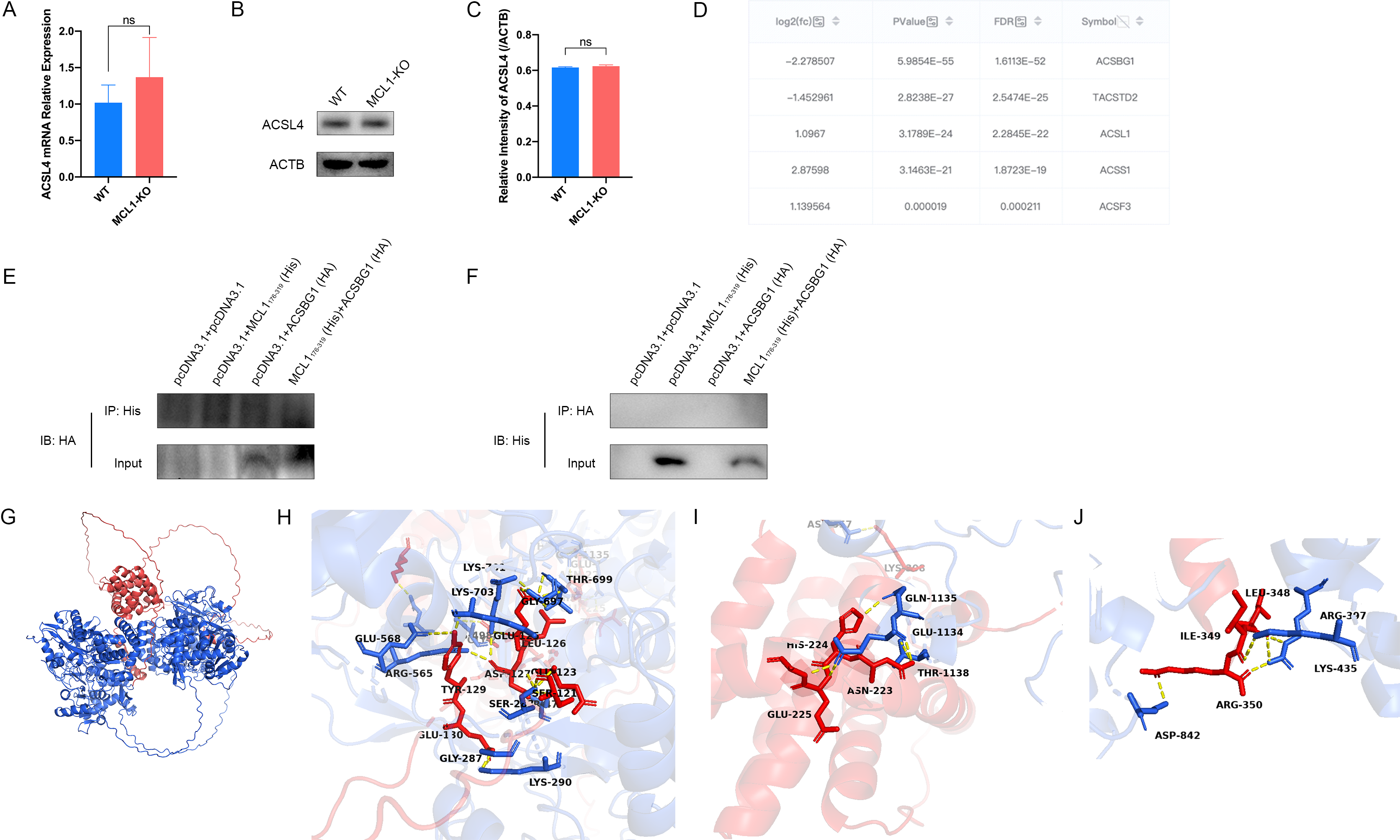

Supplement: S4 Fig — ACSL4 mRNA relative expression and protein levels in MCL1-KO and WT LLC-PK1 cells were measured by qRT-PCR (A) and western blot (B). The band intensity of ACSL4 was quantified using ImageJ software (C). DEGs in the ACS family identified from the transcriptomic dataset are shown (D). ACSBG1-HA were immunoprecipitated with anti-His binding beads (E), and MCL1176–319-His were immunoprecipitated with anti-HA binding beads (F). HEK293T cells transfected with corresponding empty vectors served as the controls. Ribbon diagram represented the structure of human-derived ACSBG1-MCL1 complex with ACSBG1 colored in blue and MCL1 in red (G). Close-up views of the interface highlighted specific residues from ACSBG1 (blue) and MCL1 (red), and the predicted interaction sites were indicated by yellow bonds (H-J). The presented results represent the means and standard deviations of the data from three independent experiments. Ns, not significant. (TIF) [file ppat.1014170.s004.tif]
