# Supplementary material for: MCL1 promotes porcine epidemic diarrhea virus replication by modulating arachidonic acid metabolic pathway
Source: PLoS Pathog. 2026 Apr 24;22(4):e1014170. doi: 10.1371/journal.ppat.1014170 (PMC13138738; doi:10.1371/journal.ppat.1014170)
Supplement: S1 Table — (DOCX) [file ppat.1014170.s005.docx]

**S1 Table**

| Gene name | Forward sequence (5’-3’) | Reverse sequence (5’-3’) |
| --- | --- | --- |
| siMCL1 | CAGUACGGAUGGGUCACUATT | UAGUGACCCAUCCGUACUGTT |
| siPLA2G5-1 | GCACGAUGAUUGAGAAGGUTT | ACCUUCUCAAUCAUCGUGCTT |
| siPLA2G5-2 | CCUUGACACAGUCGUACAATT | UUGUACGACUGUGUCAAGGTT |
| siPLA2G5-3 | CGAAACAGGAGGAGCUACATT | UGUAGCUCCUCCUGUUUCGTT |
| siACSBG1 | CAGAGAAGGUGAAGGAUGATT | UCAUCCUUCACCUUCUCUGTT |
| NC | UUCUCCGAACGUGUCACGUTT | ACGUGACACGUUCGGAGAATT |
