# Supplementary material for: MCL1 promotes porcine epidemic diarrhea virus replication by modulating arachidonic acid metabolic pathway
Source: PLoS Pathog. 2026 Apr 24;22(4):e1014170. doi: 10.1371/journal.ppat.1014170 (PMC13138738; doi:10.1371/journal.ppat.1014170)
Supplement: S2 Table — (DOCX) [file ppat.1014170.s006.docx]

**S2 Table**

| Gene name | Forward sequence (5’-3’) | Reverse sequence (5’-3’) |
| --- | --- | --- |
| pMCL1 | GGGGTACCCCATGTTCGGCCTGCAGCGCAAC | GGAATTCCTCAGTGGTGGTGATGGTGGTGGTGGTGCCGGATCAGGTAAGCCAGTCC |
| pMCL1_1-175_ | GGGGTACCCCATGTTCGGCCTGCAGCGCAAC | GGAATTCCTCAATGGTGATGGTGATGATGCAGCTCGTCCTCCTCCTCC |
| pMCL1_176-319_ | GGGGTACCCCATGTACCGGCAGAGCCTGGAGATC | GGAATTCCTCAATGGTGATGGTGATGATGGAACTCCACGAAGCCGTCCCAGC |
| pMCL1_320-351_ | GGGGTACCCCATGTTCCACGTGGAGGACCTGGAG | GGAATTCCTCAGTGGTGGTGATGGTGGTGGTGGTGCCGGATCAGGTAAGCCAGTCC |
| pACSBG1 | GGGGTACCCCGCCACCATGCCACGCAGTTCTGGACC | CGCGGATCCGCGTCAAGCGTAATCTGGAACATCGTATGGGTATTGTGGGACCCAGCTCTCCAC |
