# Supplementary material for: MCL1 promotes porcine epidemic diarrhea virus replication by modulating arachidonic acid metabolic pathway
Source: PLoS Pathog. 2026 Apr 24;22(4):e1014170. doi: 10.1371/journal.ppat.1014170 (PMC13138738; doi:10.1371/journal.ppat.1014170)
Supplement: S3 Table — (DOCX) [file ppat.1014170.s007.docx]

**S3 Table**

| Gene name | Forward sequence (5’-3’) | Reverse sequence (5’-3’) |
| --- | --- | --- |
| sg-hMCL1 | CACCGtcggactcaacctctactgt | aaacacagtagaggttgagtccgaC |
| sg-pMCL1 | CACCGtctcttcaggcggcgacgcg | aaaccgcgtcgccgcctgaagagaC |
| sg-hMCL1-seq | CAGTAAGGAGTCGGGGTCTTCC | CGCGAAGAAAAGCAGCCTCG |
| sg-pMCL1-seq | GAGGCCGTCTCTTGGCTACG | GGTACCGAGAGATAATCTCCAGGGA |
