# Supplementary material for: MCL1 promotes porcine epidemic diarrhea virus replication by modulating arachidonic acid metabolic pathway
Source: PLoS Pathog. 2026 Apr 24;22(4):e1014170. doi: 10.1371/journal.ppat.1014170 (PMC13138738; doi:10.1371/journal.ppat.1014170)
Supplement: S4 Table — (DOCX) [file ppat.1014170.s008.docx]

**S4 Table**

| Gene name | Forward sequence (5’-3’) | Reverse sequence (5’-3’) |
| --- | --- | --- |
| pMCL1 | CTGGGGCAGGATTGTGACTC | AGCCAGTCTCGTTTTGTCCT |
| PEDV N | CGCAAAGACTGAACCCACTAACTT | TTGCCTCTGTTGTTACTCGGGGAT |
| pDNM2 | CTTCCTTCCCCGAGGTTCAG | GCCTTTATTGGTCCCCGTGA |
| pCLTC | CGCTTGGCATCTACTCTCGT | TCCCATCTACACACGCGAAG |
| pEPS15 | CACCTGGGAAAAGACCCATCA | AATTGCTTGGGTCAGCCTCT |
| pCAV1 | CGAGAAGCAAGTGTATGACGC | CGTCGTTGAGATGCTTGGGA |
| pRAB5A | CCAACGGGCCAAATACTGGA | GCCCTTCACAAAACGAAGCA |
| pEEA1 | CCTGAAGGCAACCCTTGAAC | ATGGTACGTTTCGCCTCGTT |
| pRAB7A | AGGAAGAAAGTGTTGCTGAAGG | AGTCTGCTCCTATTGTCGCT |
| pVPS39 | AGATCCATGTGGTTTCCCAGTT | ATGCTCCCTTTGCCTTGGAA |
| pLAMP1 | GACGCAGAGTTCTTCCCCAG | CTGTTGCTCACGCACCTGTA |
| pPLA2G5 | GTGACTGGAAAACCTGCCCT | CCCCATGCGACTCTGTACTT |
| pCYP2C49 | CCCGACTCCTCTCTCAATTCT | CCCCATCAATTAGGGCTTCTTTTAC |
| pCYP4A24 | GACCCGTCTCCTTGATGACC | GCACTCTTCGTGCGGATAGA |
| pGPX3 | GTACGCTGGCAAATACGTCC | ATGACGAGGCCAAAGGGTTC |
| pLTC4S | TGAAGGACGAGGTGGCTCTT | TGCAGGGAGAAGTAGGCTTG |
| pIL-1β | CAGCCAGTCTTCATTGTTCAGGTT | AGATTTGCAGCTGGATGCTC |
| pTNF-α | GTCTCAAACCTCAGATAAG | GTTGTCTTTCAGCTTCAC |
| pCPT1A | TGGTGTCCAAATACCTCGCC | CCTCCGCTCGACACATACTC |
| pACSL4 | CGGTTGGTCAAGGCTATGGA | GGGTTTGGCTTGTCGTGAAC |
| pACSBG1 | CTGACCCCACCGATAACCTG | CATCCTTCTTCCCCACGACC |
| pACTB | GGACTTCGAGCAGGAGATGG | AGGAAGGAGGGCTGGAAGAG |
